# Semantically Constrained Detection of Systemic Archetype-Inspired Patterns in Idiographic Case Formulations: A Rule-Based Framework Using FCM-FRHP Functional Semantics

**DOI:** 10.3390/ejihpe16080117

**Published:** 2026-08-16

**Authors:** Carlos Hurtado-Martínez, Luis Botella, Alejandro Sanfeliciano, Ernesto Aranda-Escolástico, Luis Angel Saúl

**Affiliations:** 1 Facultad de Psicología, Universidad Nacional de Educación a Distancia (UNED), 28040 Madrid, Spain; asanfeliciano.psychlab@psi.uned.es (A.S.); lasaul@psi.uned.es (L.A.S.); 2 FPCEE Blanquerna, Universidad Ramon Llull, 08022 Barcelona, Spain; lluisbg@blanquerna.url.edu; 3 Escuela Técnica Superior de Ingeniería Informática, Universidad Nacional de Educación a Distancia (UNED), 28040 Madrid, Spain; earandae@issi.uned.es

**Keywords:** Fuzzy Cognitive Maps (FCMs), psychological case formulation, systemic archetype-inspired patterns, personal construct psychology, FCM-FRHP

## Abstract

In psychotherapy, case formulation can organize clinically relevant information into a coherent account of how psychological difficulties emerge, persist, and may change. The Personal Meaning System Fuzzy Cognitive Map (PMS-FCM) represents the client’s bipolar construct system as a weighted directed graph, whereas the FCM-FRHP (Fuzzy Cognitive Map of Human Problem Formation and Resolution) provides a professional functional reference model for problem formation and resolution. This paper proposes a semantically constrained method for identifying systemic archetype-inspired configurations in PMS-FCM representations enriched with FCM-FRHP semantics. Rather than importing classical systemic archetypes directly, the method reformulates them as configurable graph templates adapted to intrapersonal bipolar construct systems. Detection combines FCM-FRHP functional roles, predefined semantic-affinity rules and PB-based structural criteria, edge-weight thresholds, and ranking criteria. The goal is to support the traceable identification of static structures that may inform the examination of clinically relevant systemic hypotheses, without treating them as diagnoses or evidence of observed temporal dynamics. The pipeline combines property-graph querying, RDF/SHACL conformance checking, ranked materialization, and rule-based trace generation. A local language model is used only after detection and conformance checking, as a constrained graph-to-text layer grounded in graph evidence and FCM-FRHP semantics. The approach offers a formally specified and reproducible method for conducting explicit, auditable pattern-level analysis of psychological case formulations.

## 1. Introduction

### 1.1. Case Formulation and the Need for Formal Idiographic Models

Psychological case formulation is a central component of clinical practice, providing an integrative framework for understanding the origin, maintenance, and potential resolution of psychological difficulties (Eells, 2015; Kuyken et al., 2009; Persons, 2008). Although case formulation can be used within both nomothetic and idiographic traditions, the present work focuses on its idiographic use. From this perspective, case formulation does not merely summarize clinical information; it organizes it into a person-specific explanatory model that relates difficulties, maintaining processes, resources, obstacles, and therapeutic possibilities within the client’s own meaning system.

This idiographic orientation is especially relevant because psychotherapy deals with phenomena that are inherently complex. Psychological problems unfold in systems characterized by interdependence, feedback, contextuality, and non-linearity, and are therefore not easily reducible to simple linear cause–effect chains. Recent work has accordingly emphasized the value of approaches capable of representing interconnected processes and evolving patterns rather than isolated variables alone (Burger et al., 2020; Hayes & Andrews, 2020). Within this broader perspective, case formulation may benefit from formal methods capable of representing not only clinically relevant contents, but also the structured relations through which those contents organize the person’s psychological functioning.

Personal Construct Psychology (PCP) provides a particularly suitable theoretical background for this aim. PCP conceives persons as active meaning-makers whose psychological functioning is organized through systems of bipolar constructs (Kelly, 1955). These systems are idiographic, hierarchically organized, and oriented toward anticipation and change. Constructivist approaches have accordingly emphasized that psychological difficulties and therapeutic change should be understood in relation to the person’s own meanings, self-organization, and reconstruction processes (Botella & Feixas, 1998/2008; Feixas & Villegas, 1990; Mahoney, 1991). However, although PCP offers a rich conceptual framework for understanding personal meaning systems, formalizing the reciprocal, graded, and potentially circular relations among constructs requires additional representational tools.

### 1.2. Fuzzy Cognitive Maps and the Distinction Between Professional and Idiographic Representations

Fuzzy Cognitive Maps (FCMs) provide a general formalism for representing complex systems as directed graphs in which nodes denote concepts and edges denote causal or influence relations between them (Kosko, 1986, 1992). Subsequent reviews have emphasized their use as cyclic directed inference networks for knowledge representation, reasoning, and the analysis of causal complex systems (Papageorgiou & Salmeron, 2013). Their use in psychotherapy is especially relevant because they allow relations to be represented as directed, weighted, and potentially reciprocal influences, thereby accommodating feedback, graded causality, and uncertainty in psychological systems.

In the present context, however, it is important to distinguish two different uses of FCMs. The first is the use of an FCM as a professional or third-person reference model. The Fuzzy Cognitive Map of Human Problem Formation and Resolution (FCM-FRHP) represents a clinical model composed of functional factors involved in the formation and resolution of human problems. These factors include Problem, Predisposing factors, Triggers, Maintenance factors, Reconstruction process, Resources and competencies, Motivation, and Difficulties (Botella et al., 2022; Saúl et al., 2023). In this sense, FCM-FRHP is not itself the client’s idiographic map. Rather, it is a nomothetic, professional-construct map that organizes clinically relevant knowledge about therapeutic change through functional categories and theoretically specified relations among them.

The second use is idiographic and first-person. Following Saúl et al. (2023), who use the term PMS-FCM for the idiographic first-person map of the patient’s Personal Meaning System, the present study uses PMS-FCM (Personal Meaning System Fuzzy Cognitive Map) to refer to the FCM representation of the client’s own personal construct system. In this case, nodes correspond to the client’s own bipolar constructs, usually expressed in the person’s own terms, and directed weighted edges represent the implications or perceived causal relations among those constructs. This PMS-FCM does not replace the client’s language with professional categories. Instead, it formalizes the relational structure of the person’s own meaning system while preserving its idiographic specificity.

The Weighted Implication Grid (WimpGrid) provides a structured procedure for eliciting this idiographic map. Through a semi-structured interview, the person evaluates hypothetical transformations in self-perception across bipolar constructs. These anticipatory judgments are then formalized as a weighted directed graph in which constructs are represented as nodes and perceived influences among constructs as directed weighted edges (Sanfeliciano et al., 2025a). In this way, WimpGrid provides a bridge between constructivist assessment and graph-theoretical modeling of psychological change.

The distinction between FCM-FRHP and PMS-FCM is central to the present study. FCM-FRHP provides the clinical-functional semantics of problem formation and resolution. PMS-FCM provides the idiographic graph representation of the person’s construct system. In applied case formulation, both levels may be articulated: the idiographic construct map captures the client’s subjective organization, while the FCM-FRHP reference model helps interpret constructs in terms of functional roles relevant to therapeutic change.

### 1.3. The 2C3P-FCM Procedure as Broader Clinical Background

The present work is also situated within the broader clinical and procedural tradition of the 2C3P-FCM approach: A Procedure for Case Conceptualization and Psychotherapeutic Process Planning with Fuzzy Cognitive Maps of the Client’s System of Personal Meanings (Botella, 2024). This procedure proposes the use of FCMs to model the client’s personal meaning system, identify relevant components, analyze interactions and patterns, relate them to systemic archetypes, and support psychotherapy planning.

Nevertheless, the scope of the present article is more restricted. It does not aim to reproduce, replace, or extend the full 2C3P-FCM clinical protocol. The full procedure includes clinical operations such as constructing the map, identifying relevant nodes, relating them to FCM-FRHP factors, analyzing system dynamics, identifying events, patterns, structures, and mental models, selecting therapeutic goals, planning interventions, and revising the process over time (Botella, 2024). By contrast, the present study addresses a narrower methodological layer within this broader tradition: the graph-semantic detection, validation, materialization, tracing, and constrained interpretation of systemic archetype-inspired configurations in FCM-FRHP-enriched idiographic case representations.

Accordingly, the proposed pipeline should not be understood as a complete case-conceptualization protocol, but as a computational extension of one specific analytic step: the identification of higher-order systemic configurations within idiographic formulations enriched with FCM-FRHP semantics.

### 1.4. Positioning Within Contemporary Idiographic Psychological Modeling

The present framework belongs to a broader family of idiographic approaches that seek to represent psychological functioning at the level of the individual, rather than assuming that group-level relations necessarily apply to each person. These approaches include individualized behavioral assessment, personalized contemporaneous and temporal networks estimated from intensive longitudinal data, formalized dynamical case conceptualizations, and process-based idionomic assessment (Bringmann et al., 2013; Burger et al., 2020; Epskamp et al., 2018; Fisher et al., 2017; Haynes et al., 2009; Sanford et al., 2022).

These approaches differ principally in how relations are obtained and in the inferential questions they address. Personalized temporal networks use repeated observations to estimate within-person contemporaneous or lagged statistical dependencies. Formal dynamical case models may translate clinician-generated functional analyses into mathematical systems that can be used to simulate trajectories and intervention scenarios. Process-based idionomic approaches combine theory-guided process selection with intensive repeated assessment to estimate person-specific relations among processes and outcomes.

The present framework is complementary to, rather than a replacement for, these approaches. Its edges are derived from anticipated implications elicited through the WimpGrid procedure under hypothetical changes in bipolar personal constructs; they are therefore not estimates of temporal association obtained from intensive longitudinal observations. It also differs from clinician-generated cognitive-behavioral maintenance cycles represented as networks. Although both approaches formalize hypothesized maintaining relations, the present representation combines implications elicited from the person’s bipolar construct system with functional roles established through the FCM-FRHP case-formulation protocol.

The FCM-FRHP layer adds a functional reference structure in which each idiographic construct is linked to a factor established during elicitation and case formulation, such as Problem, Maintenance factors, Reconstruction process, Resources and competencies, Motivation, or Difficulties. These roles are not inferred retrospectively from construct wording or graph topology by the detection algorithm. Instead, they are encoded as part of the case representation, allowing topologically similar configurations to be distinguished according to their positions in the formation or resolution of the formulated problem.

The archetype-inspired templates also serve a different purpose from standard motif or community analyses. Topological motifs identify recurring local structures, whereas community-detection methods identify groups of relatively densely interconnected nodes. By themselves, these methods do not determine whether a configuration satisfies a specified combination of functional roles, construct polarity, edge direction, edge sign, structural thresholds, and pattern-specific eligibility conditions. The proposed templates encode these requirements explicitly and preserve a trace of the evidence supporting each detected instance. This supports theory-guided interpretation and auditability, while making the effects of template definitions and configuration parameters available for inspection and sensitivity analysis.

The framework could also benefit from future extensions addressing the correspondence between alternative or repeated case formulations. Whereas fuzzy edge weights encode the graded direction and magnitude of anticipated influence within a given map, fuzzy similarity-based comparisons could provide a complementary representation of the semantic and structural correspondence between two maps. Such comparisons could consider construct meaning, functional-factor alignment, relational profiles, structural positions, and congruence states, thereby identifying elements that are closely aligned, partially overlapping, or ambiguous across formulations. This additional layer would complement, rather than modify, the current rule-based pattern-detection procedure.

### 1.5. From Case Representation to Higher-Order Structural Organization

Once an idiographic formulation has been represented as a weighted directed graph and enriched with FCM-FRHP functional semantics, an important next step is to identify multi-node configurations that may reflect clinically meaningful systemic organization. This step is coherent with both constructivist case formulation and systems thinking. In constructivist terms, the person’s meaning system is not merely a list of constructs, but an organized network of relations in which some constructs may support, inhibit, maintain, or transform others (Botella & Feixas, 1998/2008; Kelly, 1955). In systems terms, clinical difficulties may be understood not only at the level of events or isolated symptoms, but also at the level of recurring patterns, underlying structures, and broader explanatory models (Meadows, 2008; Senge, 2006; Sterman, 2000).

Graph-based modeling provides a formal way to operationalize this movement from local relations to higher-order organization. In the present context, the relevant question is not simply whether one construct influences another construct, but whether a set of constructs and relations jointly instantiate a recognizable configuration compatible with a clinically meaningful systemic pattern. Such configurations may include reciprocal reinforcement, limiting feedback, erosion of protective processes, or competition between problem-maintaining and reconstructive loops.

However, detecting such configurations cannot be reduced to purely topological graph matching. In idiographic psychological systems, the meaning of a relation depends on construct polarity, current-self and ideal-self positioning, construct type, functional role, and the clinical semantics of the formulation. A positive edge is not inherently adaptive, nor is a negative edge inherently maladaptive. Its meaning depends on what kind of movement it implies within the bipolar construct system. For this reason, pattern detection in FCM-FRHP-enriched formulations requires a semantically constrained approach that combines graph structure with explicit psychological and functional interpretation constraints.

### 1.6. Systemic Archetypes and Their Reinterpretation in Intrapersonal Systems

Systemic archetypes offer a useful conceptual resource for this purpose. In systems thinking, archetypes such as escalation, limits to growth, drifting goals, shifting the burden, and success to the successful describe recurrent structural logics associated with characteristic patterns of system behavior over time (Meadows, 2008; Senge, 2006; Sterman, 2000). They are not merely labels for isolated events, but templates for understanding how feedback structures can generate persistent, escalating, self-limiting, or self-reinforcing patterns.

Nevertheless, their direct transfer to psychotherapy is not straightforward. Classical systemic archetypes were not originally formulated for intrapersonal idiographic cognitive systems, and their semantics cannot simply be imported unchanged. In organizational or socio-technical systems, variables are often treated as unipolar quantities whose increase or decrease has a relatively stable interpretation. In personal construct systems, by contrast, constructs are bipolar dimensions whose meaning depends on the person’s own poles, self-position, ideal-position, and anticipated direction of change.

What is needed is not the literal detection of classical systemic archetypes, but a principled reinterpretation of archetype-inspired structures within the semantic and structural conditions of FCM-FRHP-enriched case formulation. In the present work, archetype-inspired patterns are reformulated as constrained subgraphs adapted to intrapersonal cognitive systems. Causal direction, edge sign, construct polarity, self–ideal positioning, construct typology, FCM-FRHP functional role, semantic affinity with pattern families, and structural salience all contribute to admissibility and interpretation.

### 1.7. Aim and Contribution of the Study

The present study proposes a semantically constrained, graph-based methodology for characterizing and detecting systemic archetype-inspired patterns in idiographic PMS-FCM representations enriched with FCM-FRHP functional semantics. The method identifies static weighted graph configurations that are compatible with predefined archetype-inspired templates under explicit psychological, functional, semantic, and structural constraints.

The framework presupposes an idiographic FCM developed within the FCM-FRHP case-formulation protocol, in which the functional position of each construct is established during elicitation and formulation. It is not designed to infer functional-factor assignments retrospectively from independently constructed cognitive maps; the graph links computationally encode these pre-established roles.

The proposed method reformulates archetype-inspired patterns as configurable graph templates adapted to intrapersonal FCMs. Candidate eligibility is determined through multiple layers of information, including causal sign and weight, construct polarity, self–ideal positioning, construct typology, FCM-FRHP functional role, semantic compatibility with pattern families, and structural relevance derived from the Presence–Implication Balance (PB) framework (Sanfeliciano et al., 2025b). The approach does not rely on data-driven pattern discovery. Instead, it applies explicit role constraints, semantic compatibility thresholds, structural thresholds, PB-based filters, and ranking criteria to determine which candidate subgraphs are compatible with each pattern template.

Semantic compatibility parameters are encoded in the FCM-FRHP reference graph as transparent, theory-informed configuration settings for eligibility. Eligible candidates are then ranked and materialized as explicit graph instances linked to the corresponding pattern template and participating constructs. This representation makes it possible to inspect the participating constructs, their roles, the constraints satisfied, and the structural evidence supporting each materialized instance.

The contribution of this study is threefold. First, it extends the analytical capabilities of idiographic PMS-FCM representations enriched with FCM-FRHP functional semantics by introducing an explicit layer for characterizing higher-order structural organization. Second, it operationalizes this layer through a formally specified and auditable computational pipeline. The pipeline combines property-graph querying, RDF/SHACL-based rule-conformance checking, candidate ranking and materialization, and rule-based trace generation. Third, the study distinguishes two components with different methodological functions. An end-to-end illustrative synthetic vignette demonstrates the complete graph-semantic pipeline. A separate controlled synthetic rule-fidelity evaluation examines positive recovery for all four templates, rejection of a global-negative case and prespecified near-miss configurations, and transparent handling of multiple competing candidates. Automated preprocessing checks also verify the integrity of the canonical pole-orientation transformation before the case representations enter the detection pipeline.

A local language model is used only after graph-based detection and rule-conformance checking. It functions as a constrained graph-to-text layer for generating structured descriptions of already materialized pattern instances. Its input is restricted to selected graph evidence, template definitions, role assignments, FCM-FRHP semantics, and methodological constraints (Lewis et al., 2020).

This design addresses known risks of unsupported natural-language generation (Ji et al., 2023). It also reflects the need for cautious, expert-supervised use of language models in clinical contexts (Singhal et al., 2023). The language model does not participate in pattern discovery, role assignment, ranking, or rule-conformance assessment.

Accordingly, the method can identify, rank, assess the rule conformance of, and trace static graph configurations satisfying the predefined template constraints. The present study evaluates this technical feasibility and rule fidelity using controlled synthetic case representations. Specifically, it examines whether the framework recovers configurations satisfying the encoded rules, rejects negative and near-miss cases, handles competing candidates transparently, and responds predictably to selected configuration parameters.

## 2. Materials and Methods

### 2.1. Study Design and Methodological Scope

Consistent with the scope defined in Section 1.7, this methodological study combined two complementary components. First, an end-to-end illustrative synthetic case formulation was used to demonstrate the complete workflow from WimpGrid-derived case representation to pattern retrieval, ranking, rule-conformance checking, materialization, trace generation, and graph-to-text interpretation. Because this case was intentionally constructed to contain interpretable target configurations, it was treated as a worked methodological vignette rather than as an evaluation of detection performance.

Second, a separate controlled synthetic test suite was used to examine whether the implementation behaved consistently with the structural, functional, semantic, and PB-based rules encoded in the four pattern templates. This component included positive, global-negative, single-condition near-miss, and competing-candidate configurations with prespecified expected outcomes. Its purpose was to assess rule fidelity, controlled recovery and rejection behavior, and localization of implementation failures. It was not designed to estimate population-level sensitivity or specificity, false-positive or false-negative rates, or the psychological or clinical validity of the templates. The workflow combined PMS-FCM graph representation, FCM-FRHP semantic enrichment, configurable pattern templates, and PB-based structural qualification. Subsequent stages comprised Neo4j-based candidate retrieval, template-specific ranking, RDF/SHACL conformance checking, graph materialization, trace generation, and constrained graph-to-text interpretation.

**Figure 1:**
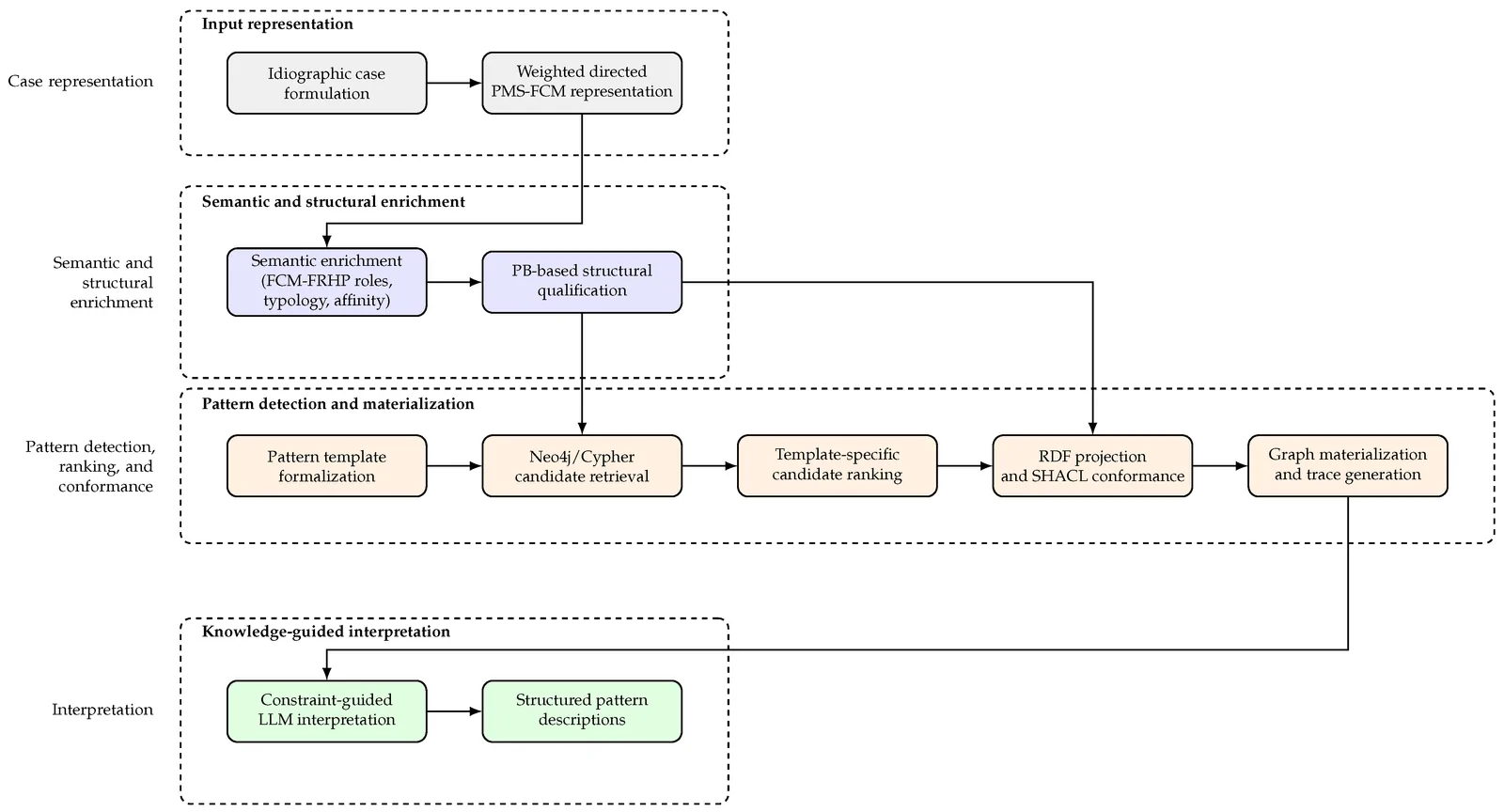
Computational pipeline for the characterization, detection, ranking, conformance checking, materialization, trace generation, and interpretation of systemic archetype-inspired patterns in FCM-FRHP-enriched idiographic PMS-FCM case representations. Arrows indicate the direction of processing flow between stages.

Case representations, reference structures, and materialized pattern instances were stored, queried, and retrieved from the graph database. Cytoscape.js 3.26.0 was used as a visualization layer to inspect and communicate these structures (Franz et al., 2016). The overall workflow is summarized in Figure 1.

### 2.2. Synthetic FCM-FRHP Case Representation

The method was illustrated using a synthetic FCM-FRHP case representation rather than empirical patient data. The case preserved relevant characteristics of idiographic formulations, including bipolar construct polarity, directed implications, functional roles, construct status, and structural heterogeneity, while avoiding the disclosure of clinically sensitive information.

The synthetic formulation was first encoded as a Weighted Implication Grid (WimpGrid), a graph-theoretical and algebraic formalization of personal construct systems designed to model psychological change through weighted directed implications (Sanfeliciano et al., 2025a). Bipolar constructs were assessed in terms of their possible implications for other constructs, producing a signed weighted matrix of perceived influences. The WimpGrid was then transformed into a fuzzy cognitive map (FCM), in which constructs were represented as nodes and weighted implications as directed edges, following the FCM formalism introduced by Kosko (1986).

After FCM construction, construct orientation was checked and, when required, normalized so that the pole aligned with the Ideal-Self was placed on the right. The synthetic case already followed this convention. Right-pole alignment was used solely as an analytical convention, with pole labels and incident edge signs transformed consistently whenever reorientation was required.

Before construct-level descriptors and graph payloads were derived, each construct with a defined Ideal-Self orientation was converted automatically to a canonical representation in which the pole aligned with the participant’s Ideal-Self position was placed on the right. Let

$$S=\mathrm{diag}(s_{1},\ldots ,s_{n}),$$

where $s_{i}=-1$ when construct *i* requires reorientation and $s_{i}=+1$ otherwise. The transformed weight matrix was computed as

$$W^{*}=SWS,$$

or, equivalently,

$$w_{ij}^{*}=s_{i}w_{ij}s_{j}.$$

The standardized self and ideal scores were transformed consistently:

$${\mathrm{self}}_{i}^{*}=s_{i}{\mathrm{self}}_{i},\;{\mathrm{ideal}}_{i}^{*}=s_{i}{\mathrm{ideal}}_{i}.$$

Thus, an edge changed sign when exactly one of its incident constructs was reoriented and retained its sign when neither or both constructs were reoriented. The import function performed explicit integrity checks to verify preservation of absolute edge magnitudes, PB descriptors, and self–ideal distances, and stopped execution if any of these properties was altered. These checks concerned the integrity of equivalent recoding and were not counted as template-level outcomes in the controlled rule-fidelity suite. More generally, let $R=\mathrm{diag}(r_{1},\ldots ,r_{n})$, with $r_{i}\in \left\{-1,1\right\}$, represent an equivalent recoding of any subset of constructs with a uniquely defined Ideal-Self orientation, such that $W_{R}=RWR$, ${\mathrm{self}}_{R}=R\mathrm{self}$, and ${\mathrm{ideal}}_{R}=R\mathrm{ideal}$. The canonicalizing matrix for the recoded representation is $S_{R}=SR$, and therefore,

$$S_{R}W_{R}S_{R}=SWS.$$

Equivalent pole recodings consequently produce the same canonical signed graph when the Ideal-Self position defines a unique pole orientation. Undefined-self constructs remain covered by this procedure because their Ideal-Self position may still identify a desired pole. Dilemmatic constructs have a central Ideal-Self position and therefore do not define a unique desired-pole orientation. The import procedure does not reorient these constructs: their original pole order and the signs of their incident relations are retained. Accordingly, the formal invariance result applies to constructs with a uniquely defined Ideal-Self orientation. If a dilemmatic construct participates in a pattern, its incident signs are interpreted relative to the recorded bipolar orientation rather than as movement toward a uniquely desired or undesired pole.

The method presupposes that the idiographic FCM has been developed within the FCM-FRHP case-formulation framework (Botella, 2024; Botella et al., 2022; Saúl et al., 2023). Within this framework, the functional factor of each construct is established during construct elicitation and case formulation and therefore constitutes part of the construct metadata before the computational pipeline is applied.

To clarify this prospective functional assignment, the synthetic formulation followed an ordered 15-position structure based on guiding questions associated with the eight FCM-FRHP factors. Position C01 corresponded to the Problem; C02–C03 to Predisposing factors; C04–C05 to Triggers; C06–C07 to Maintenance factors; C08–C09 to the Reconstruction process; C10–C11 to Resources and competencies; C12–C13 to Motivation; and C14–C15 to Difficulties. Each guiding question delimited the functional domain within which one or more bipolar constructs were specified. Functional assignments were therefore established prospectively from the construct position and elicitation context, rather than inferred retrospectively from pole wording, graph topology, or the pattern-detection algorithm. The guiding questions and their correspondence with construct positions and FCM-FRHP factors are reported in Appendix A, Table A1.

When the WimpGrid-derived FCM was incorporated into the graph database, the pre-established functional factor was preserved. Each construct node was linked to the corresponding FCM-FRHP reference-factor node. This additional semantic layer supports pattern detection in individual formulations and may also enable Graph Data Science analyses across cases or populations encoded under the same formulation and data-modeling protocol.

The framework is not intended to assign FCM-FRHP factors post hoc to constructs from independently generated fuzzy cognitive maps. In the present synthetic study, functional-factor assignments were specified prospectively by the authors through the 15-position elicitation structure and its associated guiding questions. In applied FCM-FRHP use, the client contributes the idiographic construct content, whereas the interviewer records the functional factor defined by the elicitation context. Factor assignment is therefore not inferred by the algorithm from construct wording, semantic similarity, or graph topology.

The current implementation stores one prespecified primary factor per construct. It does not represent multiple or uncertain factor memberships, and no inter-rater reliability assessment was conducted. Because factor membership constrains pattern-role eligibility, a different upstream formulation assignment could alter the candidates and patterns detected.

### 2.3. Graph Representation of the FCM-FRHP Reference Model

In addition to representing the synthetic case as an idiographic graph, the FCM-FRHP reference structure was also implemented as a computational graph. This reference structure was not introduced here as a new clinical formulation model. Rather, it operationalized, in a Neo4j property-graph environment, the functional organization already described in the FCM-FRHP clinical supervision and case-formulation literature (Botella, 2024; Saúl et al., 2023). In that framework, constructs elicited during formulation can be understood through functional factors involved in the formation and resolution of human problems, including Problem, Predisposing factors, Triggers, Maintenance factors, Difficulties, Reconstruction process, Resources and competencies, and Motivation.

In the present implementation, these functional factors were represented as reference nodes, and the synthetic constructs were encoded with links to the corresponding FCM-FRHP factors as part of the case representation. This graph-based encoding preserved the clinical logic of the FCM-FRHP model while making it available for computational operations such as candidate retrieval, semantic filtering, pattern-template matching, validation, trace generation, and pattern materialization.

This graph-based representation allowed the detection pipeline to distinguish between two levels of information: the idiographic FCM of the synthetic case and the functional reference structure used to interpret construct roles. Thus, pattern detection was not based only on local edge topology, but also on the semantic position of each construct within the FCM-FRHP framework.

In this reference graph, each FCM-FRHP factor also carried pattern-specific affinity parameters. These author-defined parameters were used as configurable settings for semantic eligibility and traceability rather than as empirical measures. Their derivation, interpretation, and complete factor-by-pattern configuration are described in Section 2.5 and Appendix B.

**Figure 2:**
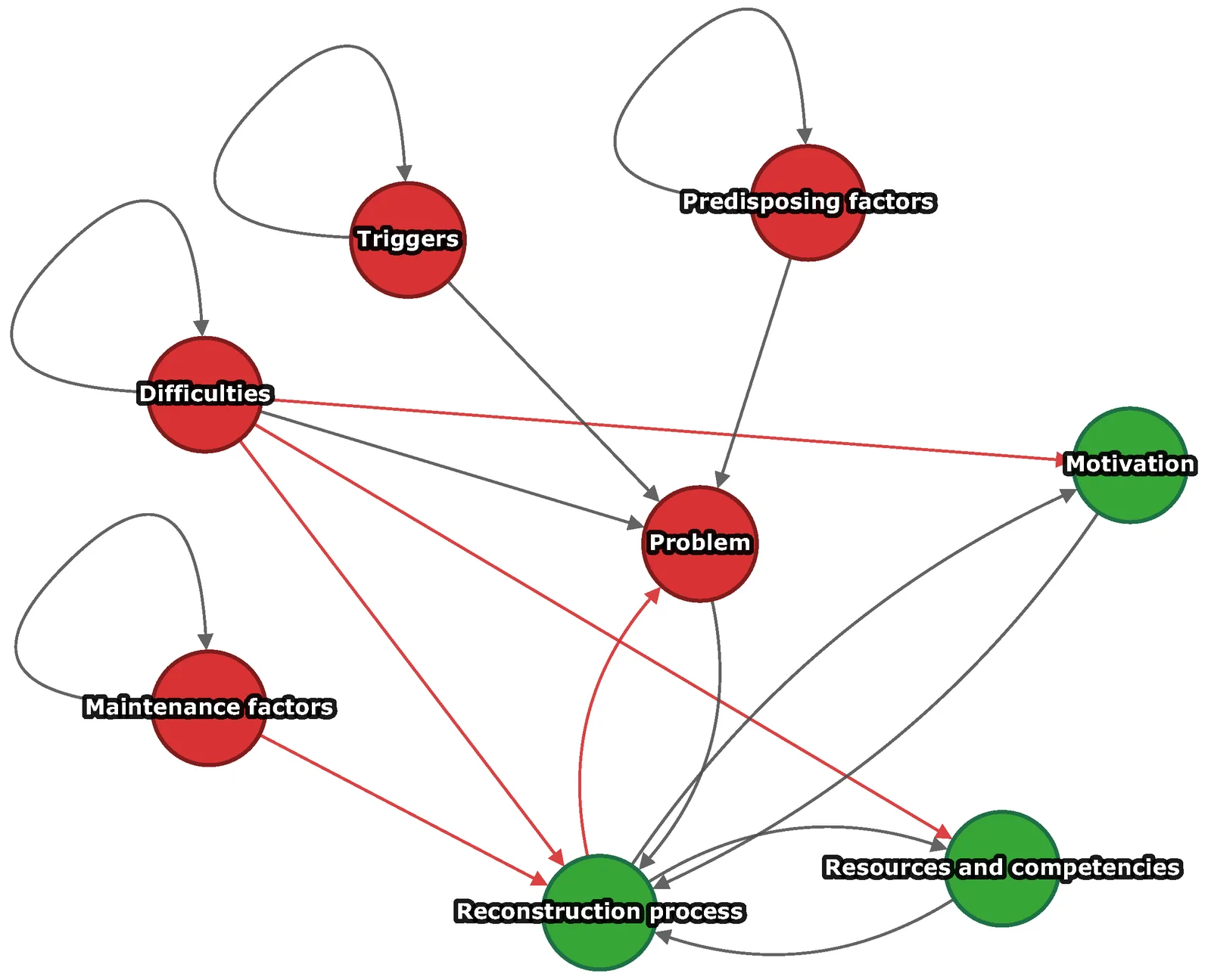
Graph-based representation of the FCM-FRHP reference model, based on Saúl et al. (2023). The model includes the main functional factors used to organize case formulations and provides the semantic substrate for role-constrained pattern detection in the present implementation. In this representation, red nodes denote factors associated with problem formation and maintenance, whereas green nodes denote factors associated with problem resolution. Gray and red arrows indicate positive and negative directed influences, respectively; arrowheads indicate direction, and self-loops represent self-influence. Node colors denote functional groupings within the reference model and do not encode the congruence states of idiographic constructs. The displayed arrows represent the literature-derived FCM-FRHP relations and are distinct from the pattern-specific affinity values used as computational criteria.

Figure 2 shows the graph-based representation of the FCM-FRHP reference model, based on Saúl et al. (2023), and used in the present study as the semantic substrate for role-constrained pattern detection.

### 2.4. Archetype-Inspired Pattern Families

In the present framework, the four archetype-inspired patterns are not interpreted as direct clinical diagnoses or as observed temporal dynamics, but as graph-semantic configurations that may organize an idiographic system of personal meanings. In all four cases, the clinical interpretation concerns potential movements implied by the signed weighted structure of the FCM, not empirically observed temporal trajectories. The templates therefore identify graph-compatible tendencies that may organize the case formulation under explicit semantic and structural constraints.

**Figure 3:**
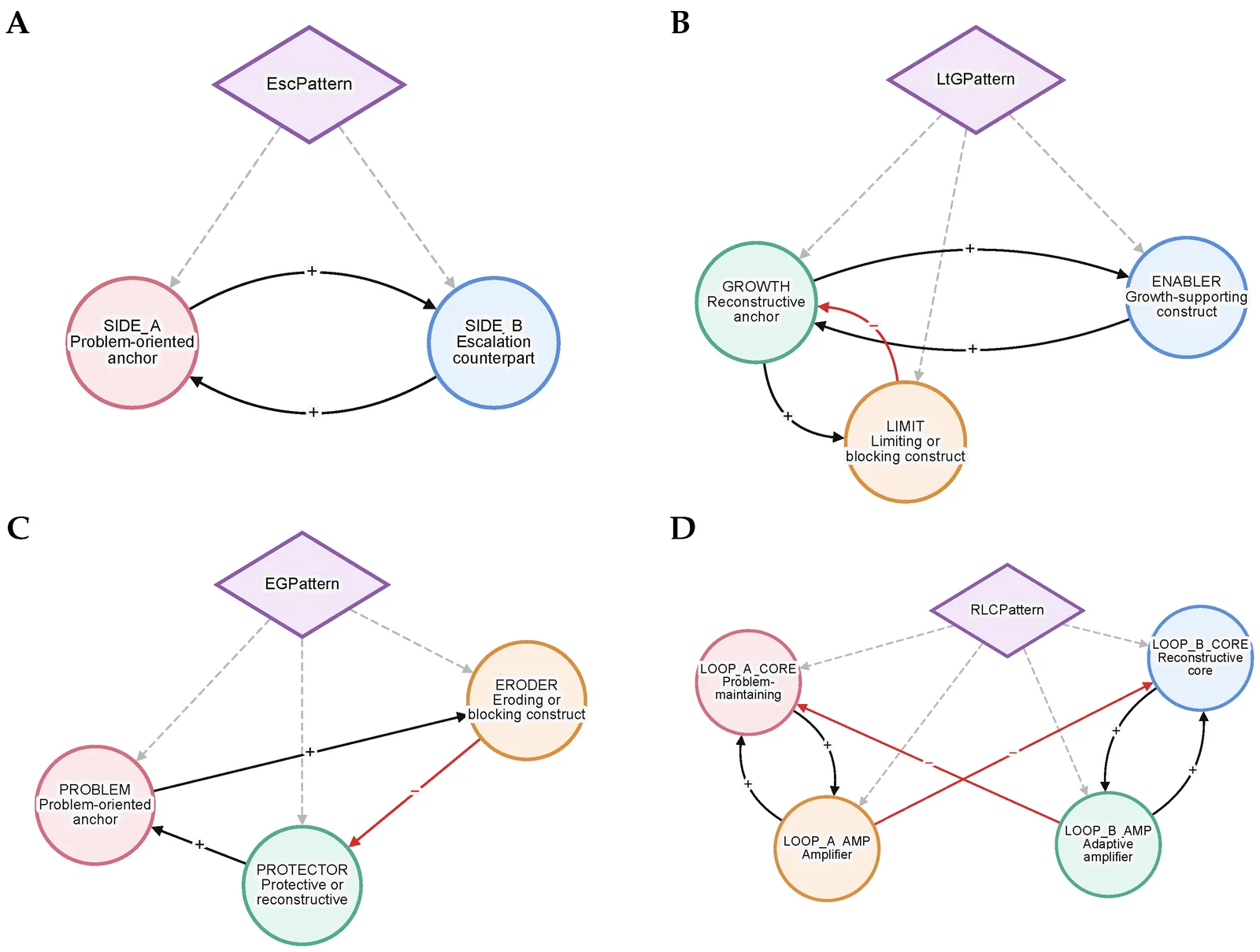
Schematic representation of the four archetype-inspired pattern templates formalized in the present study: (**A**) Escalation, (**B**) Limits to Growth, (**C**) Eroding Goals, and (**D**) Reinforcing Loops in Competition, hereafter abbreviated as Esc, LtG, EG, and RLC, respectively. Each panel represents a role-based graph template rather than a detected case-specific instance. Dashed arrows indicate template-role membership, whereas solid directed edges indicate required signed influences. Positive relations are shown in black and negative relations in red.

Figure 3 provides a compact representation of the four role-based pattern templates before their case-specific detection and materialization.

Escalation refers to a reciprocal configuration in which a problem-oriented construct and a semantically compatible counterpart are linked by same-direction implications. In the intrapersonal interpretation adopted here, this does not mean that an actual temporal escalation has been observed. Rather, the configuration indicates that a potential movement of one construct toward its undesirable pole—defined by the canonical pole-orientation procedure described in Section 2.2 as the pole opposite to the participant’s Ideal-Self-oriented pole—could pull the other construct toward its own undesirable pole, according to the anticipated implications encoded in the FCM. Thus, escalation is not understood as competition between external agents, but as a structurally compatible intrapersonal loop in which maladaptive movement in one construction may amplify the central problem, while the problem-oriented construct may reinforce the counterpart in return.

Limits to Growth captures a configuration in which a reconstruction-oriented construct and an enabling construct are linked by same-direction implications, forming a potential growth-supporting pathway, while a limiting construct is connected to this pathway through a negative feedback relation. In the interpretation adopted here, this does not mean that an actual growth process has been observed and then empirically shown to slow down. Rather, the configuration indicates that movement toward a reconstructive or desired pole could be supported by an enabling construct, but also potentially constrained by a difficulty, maintenance factor, or perceived cost of change. Clinically, the pattern points to formulations in which therapeutic change, motivation, or personal resources may be structurally present, while the graph also contains relations suggesting possible restriction of their effect.

Eroding Goals describes a configuration in which a problem-oriented construct is linked to an eroding pathway that may weaken a protective or reconstructive construct. In this interpretation, erosion is not treated as an observed temporal lowering of goals. Rather, the template identifies a potential structural route through which movement associated with the problem could undermine a construct that would otherwise support protection, reconstruction, or movement toward a desired pole. Clinically, this may correspond to formulations in which the person could accommodate the problem, reduce aspirations, or normalize a maladaptive position, not as an established longitudinal fact, but as a graph-compatible tendency suggested by the encoded implications.

Finally, Reinforcing Loops in Competition represents the coexistence of two internally reinforcing configurations: one problem-maintaining and one reconstructive or adaptive. The negative cross-relations between them indicate that movement within one loop could inhibit or reduce the viability of movement within the other. Clinically, this pattern does not demonstrate an observed oscillation or temporal dominance between loops. Rather, it identifies a structurally compatible organization in which the personal meaning system contains two mutually constraining tendencies: one that may preserve the problem-oriented organization, and another that may support reconstruction.

Taken together, the proposed templates define the specific intrapersonal operationalizations adopted in this study for the four systemic archetype-inspired patterns. They were designed to preserve the core feedback logic of each pattern while adapting it to the functional and structural characteristics of an idiographic FCM-FRHP formulation. The resulting definitions provide a coherent and fully specified basis for pattern detection. The four templates should therefore be understood as one explicit and configurable set of intrapersonal operationalizations, rather than as unique or necessary translations of the corresponding classical systemic archetypes. At the same time, the configurable architecture permits theoretically justified variants to be implemented through adjustments to individual parameters or template conditions, without redesigning the overall computational framework.

### 2.5. Pattern Template Catalog and Pattern-Affinity Parameters

The affinity configuration was developed through iterative theory-informed discussion within the author team. The team included authors with clinical, constructivist, methodological, and computational expertise. The discussion considered the FCM-FRHP functional definitions and the roles specified by the four templates.

The configuration was not obtained from empirical data, independent ratings, or blinded review. No formal inter-rater agreement coefficient was calculated. Differences in judgment were resolved through discussion in order to establish a single reproducible configuration for the present implementation.

Operationally, the numerical values encoded ordered compatibility judgments rather than interval-scaled measurements. Differences between adjacent values were not interpreted as equal quantitative units. The affinity scores were not included in the structural ranking formulas.

In the present implementation, all affinity-filtered roles used a minimum threshold of 0.50, applied jointly with role-specific allowed-factor constraints. Exact factor requirements were used for the fixed anchors: Problem for Escalation SIDE_A and Eroding Goals PROBLEM, and Reconstruction process for Limits to Growth GROWTH.

Systemic archetype-inspired patterns were represented as configurable graph templates. Each template was stored as a graph object with associated metadata, including template identifier, version, pattern family, selection method, validation method, temporal status, interpretation level, and methodological note. Pattern roles were also represented as explicit graph objects, specifying their role family, selection mode, and role-specific trace information.

This design allowed the pattern catalog to remain separate from any particular case. A detected instance was therefore not treated as an ad hoc label assigned to a group of constructs, but as a materialized instance of a predefined pattern template. The link between a detected pattern and its template was represented explicitly in the graph through an INSTANCE_OF_PATTERN_TEMPLATE relationship, allowing downstream inspection of which template, version, role definitions, and constraints had been applied.

In addition to the template catalog, semantic compatibility between FCM-FRHP functional factors and archetype-inspired pattern families was encoded in the FCM-FRHP reference graph. Each reference factor was assigned pattern-specific affinity scores, namely affinity_escalation, affinity_limits_to_growth, affinity_eroding_goals, and affinity_reinforcing_competition. These scores were defined in the interval [0,1] and represented author-defined heuristic settings indicating the degree to which a given FCM-FRHP factor was considered functionally compatible with a given pattern family in the present implementation. The values were assigned through theory-informed design judgment, considering the functional definition of each FCM-FRHP factor and its compatibility with the roles required by each template. They were not estimated or calibrated from empirical data and should not be interpreted as established semantic measurements. The complete factor-by-pattern configuration and a compact functional rationale are reported in Appendix B, Table A2.

For example, the FCM-FRHP reference node Difficulties was encoded as a problematic and blocking factor, with role_group = problematic, is_problematic = true, and is_blocking = true. It also stored high affinity values for pattern families in which limiting, eroding, or problem-maintaining functions may be expected: affinity_limits_to_growth = 1.0, affinity_escalation = 0.9, affinity_reinforcing_competition = 0.9, and affinity_eroding_goals = 0.8. This illustrates how clinical-functional semantics were made explicit and inspectable as graph properties.

These affinity values were not interpreted as empirical probabilities, psychometric scores, clinical severity indices, or diagnostic weights. Rather, they functioned as transparent configuration parameters for semantic eligibility and traceability. In the present implementation, affinity scores were used to define eligibility thresholds and to document semantic compatibility.

In the Escalation template, the problem-oriented factor was used as the SIDE_A anchor. SIDE_B eligibility was determined by the Escalation affinity of the counterpart’s FCM-FRHP factor, together with the exclusion of the anchor factor itself.

In the Limits to Growth template, the GROWTH role was anchored in the Reconstruction process factor. The ENABLER and LIMIT roles were constrained by their affinity with reconstructive-supporting and limiting or blocking processes, respectively.

In the Eroding Goals template, the PROBLEM role was anchored in the problem-oriented factor. The ERODER and PROTECTOR roles were constrained by their affinity with eroding/blocking and protective/reconstructive functions, respectively.

In the Reinforcing Loops in Competition template, role eligibility was constrained by the affinity of each factor with either problem-maintaining or reconstructive/adaptive loop membership.

Across templates, affinity scores constrained semantic eligibility. Final selection among compatible candidates was determined by structural thresholds, PB-based eligibility filters when configured, and template-specific ranking criteria.

### 2.6. Pattern Formalization as Configurable Graph Templates

In this study, systemic archetype-inspired patterns were operationalized as configurable graph templates over FCM-FRHP case representations. Each template specifies the roles, semantic-affinity constraints, structural edge constraints, and scoring rules required for detecting compatible static subgraphs.

The pattern catalog included four archetype-inspired templates: Escalation, Limits to Growth, Eroding Goals, and Reinforcing Loops in Competition. The illustrative case reported in the Results section focuses on Escalation and Limits to Growth, as these two configurations were selected to provide a clear and parsimonious demonstration of the pipeline. Although the formulation is synthetic and non-identifiable, it was designed to reflect graph-semantic configurations that may arise in clinical case-formulation work, drawing on research and supervision experience within the Constructivist Research Group at UNED (GICUNED), without being derived from, or corresponding to, any identifiable clinical case. The remaining templates are nevertheless formalized here to document the broader methodological architecture and to show how additional pattern families can be represented within the same graph-semantic framework.

Before introducing the formal notation, the four templates can be summarized in terms of their principal roles and required relations. Their intrapersonal interpretation and conceptual relationship to systemic archetypes were described in Section 2.4 and are represented schematically in Figure 3.

Escalation consists of a Problem-anchored construct and an eligible counterpart linked by positive reciprocal influences.

Limits to Growth consists of a Reconstruction-process anchor and an ENABLER forming a positive reciprocal growth-supporting dyad, together with a LIMIT construct connected through a signed limiting feedback structure.

Eroding Goals consists of a PROBLEM anchor that activates an ERODER, which negatively influences a PROTECTOR involved in the problem-related feedback structure.

Reinforcing Loops in Competition consists of two positive reciprocal microloops, one problem-maintaining and one reconstruction-oriented, connected by negative cross-relations.

The following definitions express these role-based summaries as configurable graph templates by specifying the node attributes, functional and semantic eligibility conditions, signed edge requirements, and ranking criteria that candidate subgraphs must satisfy.

Let

G=(V,E,ψ,ϕ)

denote the FCM associated with an idiographic case formulation, where *V* is the set of constructs, E⊆V×V is the set of directed influences, $\psi :V\to A_{V}$ maps each construct to a structured set of node-level attributes, and ϕ:E→[−1,1] assigns signed causal weights to directed influences. In the present context, ψ(v) does not denote a single categorical label, but the set of attributes associated with construct *v* that are relevant for construct representation, semantic interpretation, role eligibility, structural qualification, and pattern detection. These attributes may include:

1. construct-identification attributes, such as construct identifier, position, and construct label;
2. construct-level representational attributes, including bipolar pole labels and self–ideal positioning when available;
3. an FCM-FRHP functional factor, such as Problem, Predisposing factors, Triggers, Maintenance factors, Difficulties, Reconstruction process, Resources and competencies, or Motivation;
4. a construct status, namely congruent, discrepant, dilemmatic, or undefined self;
5. PB-derived structural descriptors, including Presence P(v) and Implication Balance B(v), with structural filtering based only on the derived low-presence criterion computed from P(v) (Sanfeliciano et al., 2025b).

In the present implementation, construct status was retained for description and interpretation but was not included in the template-eligibility or ranking conditions.

A pattern template is defined as

$$T_{k}=\left(R_{k},A_{k},C_{k},S_{k}\right),$$

where $R_{k}$ is the set of template roles, $A_{k}$ is the set of semantic role-affinity constraints, $C_{k}$ is the set of structural constraints, and $S_{k}$ is the scoring function used for ranking compatible candidates. Role-affinity constraints were grounded in pattern-specific affinity scores encoded in the FCM-FRHP reference graph. For a functional factor *f* and a pattern family *k*, let

$$\alpha_{k}\left(f\right)\in \left[0,1\right]$$

denote the affinity between the FCM-FRHP factor *f* and pattern family *k*. These affinity values were author-defined heuristic parameters representing semantic compatibility between FCM-FRHP functions and archetype-inspired pattern families.

A candidate subgraph

$$H=(V_{H},E_{H})$$

is considered compatible with template $T_{k}$ if its participating constructs can be assigned to the roles in $R_{k}$, satisfy the semantic role-affinity constraints in $A_{k}$, and meet all structural constraints in $C_{k}$. Among compatible candidates, the scoring function $S_{k}$ determines which candidates are retained for validation and materialization.

This formulation separates five elements that are often conflated: semantic affinity, role eligibility, structural compatibility, ranking priority, and interpretive meaning. Thus, a construct is not assigned to a role merely because it belongs to a broad functional category; it must instantiate a semantically compatible FCM-FRHP factor, participate in the required weighted and directed relations, satisfy any configured PB-based eligibility filter, and achieve sufficient ranking compatibility relative to competing candidates.

#### 2.6.1. Escalation

The Escalation template was defined over a two-role configuration:

$$R_{\mathrm{Esc}}=\left\{\mathrm{SIDE}\_A,\mathrm{SIDE}\_B\right\}.$$

SIDE_A corresponds to the problem-oriented anchor, whereas SIDE_B corresponds to the structurally selected escalation counterpart. For a candidate pair (a,b), the required edge set was

$$E_{\mathrm{Esc}}=\left\{\left(a,b\right),\left(b,a\right)\right\}.$$

Role-affinity constraints required SIDE_A to instantiate the problem-oriented FCM-FRHP anchor factor. SIDE_B was defined as a flexible counterpart role whose eligibility depended on the Escalation affinity score assigned to its FCM-FRHP factor in the reference graph. Let f(a) and f(b) denote the FCM-FRHP factors instantiated by *a* and *b*, respectively. SIDE_A was required to satisfy:

f(a)=Problem.

SIDE_B was required to instantiate a non-anchor factor with sufficient Escalation affinity:

$$f\left(b\right)\neq f\left(a\right),\;\alpha_{\mathrm{Esc}}\left(f\left(b\right)\right)\geq \lambda_{\mathrm{Esc}}.$$

Here, $\alpha_{\mathrm{Esc}}\left(f\left(b\right)\right)$ denotes the affinity of factor f(b) with the Escalation pattern family, and $\lambda_{\mathrm{Esc}}$ is the configured minimum affinity threshold. In the implementation used here, SIDE_B candidates were also excluded if they were flagged by the operational low-*P* exclusion criterion.

Structurally, both directed relations were required to exceed the minimum edge-weight threshold:

$$\phi \left(a,b\right)\geq \tau_{w},\;\phi \left(b,a\right)\geq \tau_{w}.$$

The reciprocal strength of the candidate pair was computed as

$${\mathrm{strength}}_{\mathrm{Esc}}=\sqrt{\phi (a,b)\phi (b,a)}.$$

The reciprocity balance was computed as

$${\mathrm{balance}}_{\mathrm{Esc}}=1-\frac{\left|\phi (a,b)-\phi (b,a)\right|}{\phi (a,b)+\phi (b,a)}.$$

Candidate pairs were additionally required to satisfy template-level thresholds for reciprocal structural strength and reciprocity balance:

$${\mathrm{strength}}_{\mathrm{Esc}}\geq \tau_{s},\;{\mathrm{balance}}_{\mathrm{Esc}}\geq \tau_{b}.$$

The final ranking score was defined as

$$S_{\mathrm{Esc}}={\mathrm{strength}}_{\mathrm{Esc}}\times {\mathrm{balance}}_{\mathrm{Esc}}.$$

Thus, Escalation was operationalized as a positive reciprocal dyad linking a problem-oriented construct with a semantically compatible non-anchor counterpart. Candidate selection combined semantic affinity, edge-weight thresholds, reciprocal structural strength, reciprocity balance, PB-based exclusion filtering, and dyadic ranking.

#### 2.6.2. Limits to Growth

The Limits to Growth template was defined over three roles:

$$R_{\mathrm{LtG}}=\left\{\mathrm{GROWTH},\mathrm{ENABLER},\mathrm{LIMIT}\right\}.$$

GROWTH corresponds to the reconstruction-oriented anchor, ENABLER to a construct participating in the positive growth engine, and LIMIT to a construct that is activated by GROWTH and negatively feeds back into it. For a candidate triad (g,e,l), the required edge set was

$$E_{\mathrm{LtG}}=\left\{\left(g,e\right),\left(e,g\right),\left(g,l\right),\left(l,g\right)\right\}.$$

Role-affinity constraints required GROWTH to instantiate the Reconstruction process factor:

f(g)=Reconstructionprocess.

ENABLER candidates were restricted to constructs instantiating Resources and competencies, Motivation, or Reconstruction process, whereas LIMIT candidates were restricted to Difficulties or Maintenance factors. For both roles, eligibility additionally required the corresponding Limits to Growth affinity to meet the configured threshold:

$$\alpha_{\mathrm{LtG}}\left(f\left(e\right)\right)\geq \lambda_{E},\;\alpha_{\mathrm{LtG}}\left(f\left(l\right)\right)\geq \lambda_{L},$$

Thus, membership in the role-specific factor set and satisfaction of the corresponding affinity threshold were jointly required; pattern-specific affinity was necessary but not sufficient for role eligibility.

The positive growth engine required:

$$\phi \left(g,e\right)\geq \tau_{w},\;\phi \left(e,g\right)\geq \tau_{w}.$$

The limiting feedback mechanism required:

ϕ(g,l)>0,ϕ(l,g)<0,

$$\left|\phi \left(g,l\right)\right|\geq \tau_{w},\;\left|\phi \left(l,g\right)\right|\geq \tau_{w}.$$

The enabler strength was computed as

$${\mathrm{strength}}_{E}=\sqrt{\phi (g,e)\phi (e,g)},$$

and the enabler balance as

$${\mathrm{balance}}_{E}=1-\frac{\left|\phi (g,e)-\phi (e,g)\right|}{\phi (g,e)+\phi (e,g)}.$$

The limit strength was computed as

$${\mathrm{strength}}_{L}=\sqrt{\phi (g,l)|\phi (l,g)|},$$

and the limit balance as

$${\mathrm{balance}}_{L}=1-\frac{\left||\phi (g,l)|-|\phi (l,g)|\right|}{\left|\phi (g,l)|+|\phi (l,g)\right|}.$$

The final ranking score was defined as

$$S_{\mathrm{LtG}}={\mathrm{strength}}_{E}\times {\mathrm{balance}}_{E}\times {\mathrm{strength}}_{L}\times {\mathrm{balance}}_{L}.$$

Thus, Limits to Growth was operationalized as the coexistence of a positive reciprocal growth engine and a limiting feedback mechanism, with final candidate selection determined by semantic affinity, role-specific functional constraints, edge thresholds, PB-based filtering of the GROWTH anchor, and triadic ranking.

#### 2.6.3. Eroding Goals

The Eroding Goals template was defined over three roles:

$$R_{\mathrm{EG}}=\left\{\mathrm{PROBLEM},\mathrm{ERODER},\mathrm{PROTECTOR}\right\}.$$

PROBLEM corresponds to the problem-oriented anchor, ERODER to a construct activated by the problem and capable of weakening a protective or reconstructive process, and PROTECTOR to a construct that buffers or supports movement away from the problem-oriented state. For a candidate triad (p,e,g), the required edge set was

$$E_{\mathrm{EG}}=\left\{\left(p,e\right),\left(e,g\right),\left(g,p\right)\right\}.$$

Role-affinity constraints required PROBLEM to instantiate the problem-oriented FCM-FRHP anchor factor:

f(p)=Problem.

ERODER candidates were required to instantiate Problem, Maintenance factors, or Difficulties. PROTECTOR candidates were required to instantiate Reconstruction process, Motivation, or Resources and competencies. For both non-anchor roles, eligibility required membership in the corresponding role-specific factor set and an Eroding Goals affinity of at least 0.50. Pattern-specific affinity was therefore necessary but not sufficient for role eligibility. In affinity terms, this was represented as:

$$\alpha_{\mathrm{EG}}\left(f\left(e\right)\right)\geq \lambda_{\mathrm{ER}},\;\alpha_{\mathrm{EG}}\left(f\left(g\right)\right)\geq \lambda_{\mathrm{PR}},$$

together with role-specific functional constraints distinguishing eroding from protective positions.

The required structural sequence was:

$$\phi \left(p,e\right)\geq \tau_{w},\;\phi \left(e,g\right)\leq -\tau_{w},\;\phi \left(g,p\right)\geq \tau_{w}.$$

Thus, the problem-oriented construct activates an eroding construct, the eroding construct weakens the protective or reconstructive construct, and the protective construct remains positively related to the problem-oriented node.

In the current implementation, the structural strength of a candidate triad was computed as

$$S_{\mathrm{EG}}=\phi \left(p,e\right)\times \left|\phi \left(e,g\right)\right|\times \phi \left(g,p\right).$$

Candidates were ranked according to this score after satisfying semantic-affinity, role-specific, and edge-threshold constraints. PB information could be used as a posterior structural qualifier, but it was not part of the core initial detection rule for this template, since low-presence constructs may still be clinically informative within eroding pathways.

Thus, Eroding Goals was operationalized as a directed triadic configuration in which a problem-oriented construct activates an eroding pathway that weakens a protective or reconstructive construct, while the latter remains structurally connected to the problem-oriented side of the formulation.

#### 2.6.4. Reinforcing Loops in Competition

The Reinforcing Loops in Competition template was defined over four roles:

$$R_{\mathrm{RLC}}=\left\{\mathrm{LOOP}\text{\_}A\text{\_}\mathrm{CORE},\mathrm{LOOP}\text{\_}A\text{\_}\mathrm{AMPLIFIER},\mathrm{LOOP}\text{\_}B\text{\_}\mathrm{CORE},\mathrm{LOOP}\text{\_}B\text{\_}\mathrm{AMPLIFIER}\right\}.$$

LOOP_A represents a problem-maintaining reinforcing loop, whereas LOOP_B represents a reconstructive or adaptive reinforcing loop. For a candidate quartet $\left(a_{1},a_{2},b_{1},b_{2}\right)$, the required edge set was

$$E_{\mathrm{RLC}}=\left\{\left(a_{1},a_{2}\right),\left(a_{2},a_{1}\right),\left(b_{1},b_{2}\right),\left(b_{2},b_{1}\right),\left(a_{2},b_{1}\right),\left(b_{2},a_{1}\right)\right\}.$$

LOOP_A_CORE and LOOP_A_AMPLIFIER were restricted to constructs instantiating Problem, Maintenance factors, or Difficulties, representing problem-maintaining or blocking organization. LOOP_B_CORE and LOOP_B_AMPLIFIER were restricted to Reconstruction process, Resources and competencies, or Motivation, representing reconstructive or adaptive organization. In addition, the participating factors were required to satisfy the corresponding Reinforcing Loops in Competition affinity thresholds:

$$\alpha_{\mathrm{RLC}}\left(f\left(a_{1}\right)\right)\geq \lambda_{A},\;\alpha_{\mathrm{RLC}}\left(f\left(a_{2}\right)\right)\geq \lambda_{A},$$

$$\alpha_{\mathrm{RLC}}\left(f\left(b_{1}\right)\right)\geq \lambda_{B},\;\alpha_{\mathrm{RLC}}\left(f\left(b_{2}\right)\right)\geq \lambda_{B},$$

Taken together, these criteria required both membership in the corresponding role-specific factor set and satisfaction of the affinity threshold; pattern-specific affinity alone was not sufficient for eligibility.

The two internal reinforcing loops required:

$$\phi \left(a_{1},a_{2}\right)\geq \tau_{w},\;\phi \left(a_{2},a_{1}\right)\geq \tau_{w},$$

$$\phi \left(b_{1},b_{2}\right)\geq \tau_{w},\;\phi \left(b_{2},b_{1}\right)\geq \tau_{w}.$$

The cross-loop inhibition required:

$$\phi \left(a_{2},b_{1}\right)\leq -\tau_{w},\;\phi \left(b_{2},a_{1}\right)\leq -\tau_{w}.$$

The strength of the problem-maintaining loop was computed as

$${\mathrm{strength}}_{A}=\sqrt{\phi \left(a_{1},a_{2}\right)\phi \left(a_{2},a_{1}\right)},$$

and its reciprocity balance as

$${\mathrm{balance}}_{A}=1-\frac{|\phi \left(a_{1},a_{2}\right)-\phi \left(a_{2},a_{1}\right)|}{\phi \left(a_{1},a_{2}\right)+\phi \left(a_{2},a_{1}\right)}.$$

The strength of the reconstructive or adaptive loop was computed as

$${\mathrm{strength}}_{B}=\sqrt{\phi \left(b_{1},b_{2}\right)\phi \left(b_{2},b_{1}\right)},$$

and its reciprocity balance as

$${\mathrm{balance}}_{B}=1-\frac{|\phi \left(b_{1},b_{2}\right)-\phi \left(b_{2},b_{1}\right)|}{\phi \left(b_{1},b_{2}\right)+\phi \left(b_{2},b_{1}\right)}.$$

The reciprocal cross-inhibition strength was computed from the magnitudes of the two inhibitory cross-loop relations:

$${\mathrm{strength}}_{AB}=\sqrt{|\phi \left(a_{2},b_{1}\right)|\;|\phi \left(b_{2},a_{1}\right)|}.$$

The cross-inhibition balance was computed as

$${\mathrm{balance}}_{AB}=1-\frac{||\phi \left(a_{2},b_{1}\right)|-|\phi \left(b_{2},a_{1}\right)\left|\right|}{|\phi \left(a_{2},b_{1}\right)|+|\phi \left(b_{2},a_{1}\right)|}.$$

A combined ranking score could then be defined as

$$S_{\mathrm{RLC}}={\mathrm{strength}}_{A}\times {\mathrm{balance}}_{A}\times {\mathrm{strength}}_{B}\times {\mathrm{balance}}_{B}\times {\mathrm{strength}}_{AB}\times {\mathrm{balance}}_{AB}.$$

Thus, Reinforcing Loops in Competition was operationalized as two internally reinforcing microloops, one problem-maintaining and one reconstructive or adaptive, linked through reciprocal cross-inhibition. Candidate ranking combined the strength and balance of each internal loop with the strength and balance of the cross-loop inhibitory relations. Semantic affinity constrained role eligibility, whereas PB information may be used after detection as a structural qualification criterion.

#### 2.6.5. Operational Interpretation

Under this formalization, each pattern is specified as a configurable graph template grounded in systemic theory but adapted to the semantics of intrapersonal bipolar construct systems. Detection therefore consists of identifying candidate subgraphs

H⊆G

such that

$$H⊧T_{k}$$

for some template $T_{k}$, and then ranking compatible candidates according to $S_{k}$. Compatibility requires that participating constructs satisfy semantic-affinity constraints, role-specific functional constraints, structural edge constraints, and any PB-based eligibility filters configured for the template. The resulting instances are treated as structurally compatible and semantically constrained candidates that can be validated, materialized, traced, and interpreted within the FCM-FRHP framework.

Template compatibility denotes conformance with the encoded static graph conditions; it does not establish that the corresponding temporal systemic process has occurred. Claims about behavior over time require longitudinal or repeated FCM-FRHP assessments.

### 2.7. PB-Based Structural Qualification

Information from the Presence–Implication Balance (PB) space was used as a structural qualification layer within the pattern-detection process (Sanfeliciano et al., 2025b). Presence *P* summarizes the overall implication level associated with each construct within the graph, whereas Implication Balance *B* represents the asymmetry between outgoing and incoming implications. In the present study, Presence was used as the template-specific structural eligibility variable. Implication Balance was retained for the two-dimensional structural description and visualization of PB space, although it was not used as an eligibility or ranking criterion in the present templates.

Constructs with very low Presence participate less strongly in the implication structure, as operationalized by the *P* metric. Accordingly, allowing a very-low-*P* construct to occupy a pattern role subject to structural-salience filtering may reduce the salience of the resulting configuration within the organization of the map as a whole. The low-*P* filter was therefore introduced as a configurable structural-salience constraint. It was not interpreted as an empirical, clinical, psychometric, or diagnostic threshold. In particular, low Presence does not necessarily imply low psychological importance or limited therapeutic relevance.

The low-*P* criterion was defined idiographically within each PMS-FCM. Constructs were evaluated relative to the Presence distribution of the same map rather than against an external or population-level reference. In the nominal configuration, construct $v_{i}$ was classified as low in Presence when

(1) $$P_{i}<P_{\mathrm{cut}},$$

where

(2) $$P_{\mathrm{cut}}=\bar{P}-s_{P},$$

and $\bar{P}$ and $s_{P}$ denote, respectively, the within-map mean and sample standard deviation of Presence. This criterion was selected as a transparent heuristic for identifying constructs located in the lower portion of the case-specific Presence distribution.

The computation of $P_{\mathrm{cut}}$ does not require the Presence values to follow a normal distribution. Normality is relevant only to the distributional interpretation of the cutoff: under a normal model, a value one standard deviation below the mean corresponds approximately to the lower 15.87% tail,

(3) Pr(Z≤−1)≈0.1587.

For the illustrative case, the shape of the within-map Presence distribution was explored descriptively using the Shapiro–Wilk test. Given the small number of constructs, a non-significant result was interpreted only as limited descriptive evidence that the observed values were compatible with a normal-shaped distribution. Throughout the analysis, the cutoff was treated as a configurable structural-salience parameter rather than as an empirically calibrated clinical or psychometric threshold.

#### Sensitivity Analysis of Principal Configuration Parameters

A local one-factor-at-a-time (OFAT) sensitivity analysis was conducted to examine the dependence of candidate compatibility and selection on the principal configuration parameters. Each parameter was varied while all remaining template settings were held at their reference values. The audit covered all four templates, although interpretation of selection stability was restricted to scenarios in which candidates reached the decision stage affected by the varied parameter.

For the two templates materialized in the illustrative PMS-FCM, the minimum edge-weight and strength thresholds were varied over 0.25, 0.30, and 0.35. Balance thresholds were varied over 0.60, 0.70, 0.80, and 0.90, and factor-affinity thresholds over 0.40, 0.50, and 0.60. PB-based sensitivity was examined using

$$P_{\mathrm{cut}}\left(\kappa \right)=\bar{P}-\kappa s_{P}$$

with κ=0.5, 1.0, and 1.5, together with a condition in which the PB filter was disabled. Candidate retention was compared between the reference top-*k* configuration and retention of all compatible candidates. Multiplicative ranking was also compared with arithmetic-mean and minimum-component aggregation.

For each scenario, the audit recorded candidate counts at the topological, functional-role, affinity, signed-edge, edge-weight, strength, balance, PB-eligibility, compatibility, ranking, and retention stages. When the reference selection was non-empty, the selected set was compared with the reference configuration through selection preservation and Jaccard similarity. Ranking-formula comparisons were considered informative only when more than one compatible candidate reached the ranking stage, because changing a scoring formula cannot alter selection when only one candidate remains. Similarly, top-*k* was treated as a prioritization rule rather than as a criterion redefining template compatibility. These analyses assessed local sensitivity to the selected design settings and were not intended to provide empirical or clinical calibration of the parameters.

### 2.8. Graph-Based Detection, Ranking, and Conformance-Checking Pipeline

Pattern detection was implemented as a multi-stage graph-semantic pipeline. As shown in Figure 1, the workflow begins with a WimpGrid-derived FCM-FRHP case representation, proceeds through semantic and structural enrichment, retrieves compatible candidates through graph queries, ranks candidates using template-specific scoring functions, checks the selected candidates for conformance with the RDF/SHACL rules, materializes detected patterns as graph objects, and finally generates constrained natural-language interpretations.

#### 2.8.1. Candidate Retrieval in Neo4j

The first stage consisted of retrieving structurally and semantically compatible candidates from the case representations stored in Neo4j. Within this property-graph representation, each case was represented as an FCM composed of construct nodes and directed weighted INFLUENCES relations. Each construct was also linked to its FCM-FRHP functional factor through its corresponding reference node.

Candidate subgraphs were selected through declarative Cypher queries encoding the core topological, causal, and semantic constraints of each pattern template. These constraints included directionality, edge sign, minimum absolute weight thresholds, FCM-FRHP role compatibility, pattern-specific affinity thresholds, and PB-based eligibility restrictions when specified by the template. Pattern-specific affinity values were retrieved from the FCM-FRHP reference graph and used as configurable semantic eligibility parameters. This stage functioned as a theory-driven prefiltering step: only candidates satisfying the main structural and semantic conditions were retained for scoring and validation.

#### 2.8.2. Candidate Scoring and Ranking

Retrieved candidates were not treated as equivalent. Each pattern template included a scoring function designed to rank compatible candidates according to structural strength and balance after semantic-affinity and eligibility constraints had been applied. In the present implementation, affinity scores were used primarily as semantic eligibility and traceability parameters rather than as clinical weights or severity scores.

For Escalation, candidate pairs were ranked using the product of reciprocal strength and reciprocity balance. For Limits to Growth, candidate triads were ranked by combining the strength and balance of the positive growth engine with the strength and balance of the limiting feedback mechanism. Equivalent scoring logic was used for the remaining templates according to their required structural components.

The ranking process was applied in two steps. First, admissible candidates were ranked within the relevant anchor context, such as per problem-oriented anchor in Escalation or per reconstruction-process anchor in Limits to Growth. Second, the configured template-specific top-*k* parameters determined which ranked candidates proceeded to RDF/SHACL conformance checking and materialization. Top-*k* was treated as a configurable prioritization and output-management parameter rather than as a criterion of template compatibility. Candidates not selected under the configured limit remained available in the operational traces together with their scores and terminal decision reasons. The values k=1 used in the illustrative analysis were demonstration-specific settings and should not be interpreted as usual or clinically preferred defaults; larger values or retention of all admissible candidates may be configured when several structures associated with the same anchor are analytically relevant. Score ties are possible in principle, although none occurred in the analyses reported here.

To make the illustrative implementation reproducible, Table 1 summarizes the main operational parameters retrieved from the pattern-template nodes and used in the synthetic case analysis. These values were stored as template properties in the graph database and loaded at runtime before candidate retrieval, ranking, RDF/SHACL conformance checking, and materialization. They should be understood as transparent configuration settings for the methodological demonstration, not as empirically calibrated clinical thresholds.

#### 2.8.3. RDF Projection and SHACL-Based Conformance Checking

Selected candidates were projected into a lightweight Resource Description Framework (RDF) representation (World Wide Web Consortium (W3C), 2014) using rdflib. Constructs were encoded as RDF resources and the relevant relations between them were re-expressed as typed predicates corresponding to the structural logic of each pattern template. Pattern vocabularies and SHACL shapes were encoded in RDF 1.1 using the Turtle syntax and parsed programmatically (format = "turtle") before validation.

Candidate RDF graphs were then processed using pySHACL and Shapes Constraint Language (SHACL) rules (World Wide Web Consortium (W3C), 2017). SHACL was used not only for validation, but also for controlled generation of normalized pattern instances. Each pattern template was associated with a sh:NodeShape containing rule-based conditions that generated a pattern instance when the projected candidate satisfied the required structural and semantic configuration. This stage verified the structural–semantic admissibility of the candidate and produced a normalized representation of the detected pattern.

The SHACL layer was introduced to separate efficient graph retrieval from the declarative specification of pattern conformance. Cypher was used for candidate search, eligibility filtering, and ranking within Neo4j, whereas SHACL provided a machine-readable and storage-independent representation of the conditions that a projected candidate had to satisfy before materialization. It also supported the controlled generation of normalized pattern instances and consistency checking between the Neo4j and RDF representations. When the Cypher and SHACL stages encode equivalent template conditions, a correctly retrieved and projected candidate is expected to conform; disagreement may reveal inconsistencies in rule implementation, RDF projection, data typing, or template versioning. SHACL therefore formalizes the final conformance contract applied to the selected candidate. Its conformity result establishes compliance with the encoded template and data-shape requirements, rather than substantive psychological or clinical validation. This standards-based separation is consistent with the broader knowledge-graph perspective, in which schemas, query and validation languages, identity, and context make structured knowledge explicit, inspectable, and computationally actionable (Hogan et al., 2021).

#### 2.8.4. Pattern Materialization and Trace Generation

SHACL-conformant pattern instances were materialized back into Neo4j as explicit graph nodes. Each instance was linked to its corresponding pattern template through an INSTANCE_OF_PATTERN_TEMPLATE relationship, and participating constructs were connected to the pattern through role-specific relationships such as ROLE_SIDE_A, ROLE_SIDE_B, ROLE_GROWTH, ROLE_ENABLER, and ROLE_LIMIT.

To support reproducibility and auditability, materialized instances were assigned stable identifiers derived from the participating constructs. They also stored rule-application traces documenting the structural conditions, FCM-FRHP role constraints, semantic-affinity checks, PB-based eligibility filters, ranking criteria, validation status, and methodological notes. Thus, each detected pattern remained linked to the evidence and rules supporting its retrieval, conformance checking, ranking, and materialization; this attached evidence documents its rule-based derivation and does not constitute independent psychological or clinical validation.

### 2.9. Controlled Synthetic Rule-Fidelity Evaluation

A controlled synthetic test suite was specified separately from the end-to-end illustrative case. Its purpose was to determine whether the implementation produced the prespecified outcomes when individual template conditions were satisfied or violated. The suite comprised ten prespecified test specifications distributed across nine synthetic PMS-FCM representations. Because the global-negative case was evaluated independently against each of the four templates, these specifications yielded 13 template-level expected outcomes.

The controlled representations were constructed as complete synthetic WimpGrids following the ordered functional structure of an FCM-FRHP case formulation, rather than as isolated graph motifs. Each WimpGrid contained 15 bipolar constructs, Self-Now and Ideal-Self scores, hypothetical-change ratings, and prespecified FCM-FRHP functional-factor positions. Because the controlled fixtures were intended to evaluate graph-structural and rule-level behavior rather than the psychological interpretation of particular construct contents, their construct poles were assigned neutral generic labels, such as C01_L and C01_R. This preserved the bipolar structure required by the WimpGrid representation while ensuring that pattern eligibility depended on the encoded relations and FCM-FRHP functional roles rather than on the verbal content of the pole labels. The controlled fixtures preserved the same prespecified 15-position construct-to-factor mapping used in the illustrative synthetic formulation. Generic pole labels replaced case-specific verbal content, but the functional position assigned to each construct remained unchanged and was encoded before WimpGrid transformation, graph loading, and pattern detection.

The corresponding signed weighted PMS-FCMs were generated automatically using the same WimpGrid transformation, canonical pole-orientation, PB-computation, and graph-loading procedures applied to the illustrative case. All test conditions were therefore encoded at the WimpGrid-input level; no construct nodes, directed influences, edge signs, edge weights, or functional-factor assignments were manually edited in Neo4j after import. To avoid confounding the intended rule manipulations with marked differences in graph size or sparsity, the synthetic cases were designed with broadly comparable numbers of non-zero directed relations, thereby providing similar relational complexity while varying the specific positive, negative, near-miss, or competing-candidate condition under examination.

All complete synthetic WimpGrids, their derived PMS-FCM representations, the case and test manifests, prespecified expected outcomes, intermediate traces, and observed results are publicly available in the Zenodo reproducibility package (Hurtado-Martínez et al., 2026).

The suite included four positive recovery configurations, one for each of the Escalation, Limits to Growth, Eroding Goals, and Reinforcing Loops in Competition templates. It also included a global-negative PMS-FCM that was processed independently by all four template pipelines and was expected to produce no admissible or materialized instance.

Four single-condition near-miss configurations were constructed for the Escalation template. Its compact reciprocal structure permitted the effect of individual rules to be isolated while retaining the remaining conditions as far as possible. The near misses respectively contained: (a) incompatible edge signs; (b) insufficient reciprocity balance despite adequate reciprocal strength; (c) an ineligible FCM-FRHP factor in the problem-anchor role; and (d) a counterpart excluded by the configured low-P criterion. Candidate-level diagnostic traces were generated so that rejection could be localized to the corresponding signed-edge, structural-balance, functional-role, or PB-eligibility stage.

A further Escalation case contained five admissible candidates linked to the same problem-oriented anchor. This fixture was used to examine whether all admissible candidates remained traceable, whether their ranking followed the configured scoring function, and whether the top-*k* rule selected only the highest-ranked candidate for materialization without removing the remaining candidates from the audit trace.

Expected and observed outcomes were compared at the stages of candidate eligibility, structural and semantic admissibility, ranking and selection, SHACL conformance, and graph materialization. All test representations were processed through the same import, enrichment, retrieval, ranking, RDF/SHACL, and materialization procedures used for the illustrative case. The evaluation was designed to assess controlled implementation fidelity and failure behavior. It did not estimate detection-performance parameters in a representative population of synthetic or empirical cases.

### 2.10. Knowledge-Guided Interpretation with Local Large Language Models

After graph-based detection, ranking, rule-conformance checking, and materialization, a locally deployed language model was used solely as a constrained graph-to-text layer. Each prompt was assembled from selected evidence retrieved from the materialized pattern instance and the FCM-FRHP reference graph, including template definitions, role assignments, functional semantics, signed weighted relations, structural metrics, and methodological constraints. The language model did not define templates, retrieve or rank candidates, assign roles, compute PB indices, or assess rule conformance.

The graph-to-text component underwent formative author review during prompt development, but its narrative fidelity was not evaluated through an independent, blinded, or reliability-based assessment. Future studies should examine the generated interpretations using prespecified criteria, independent clinical raters, and inter-rater agreement measures.

The exact model and software versions, inference parameters, complete prompt specification, post-processing rules, and generated outputs are documented in the publicly archived reproducibility package (Hurtado-Martínez et al., 2026).

### 2.11. Software Environment, GenAI Use, and Reproducibility Disclosure

The computational workflow was implemented using a mixed R 4.5.1 and Python 3.12.8 environment. RStudio 2026.05.0 and R Markdown 2.29 were used to load the WimpGrid-derived data, conduct initial exploratory analyses of the synthetic case representation, inspect construct-level information and the Presence distribution, and load the WimpGrid-derived case data into the Neo4j 5.26.19 graph database. Python 3.12.8, in an environment managed with conda 26.1.1, was used for graph processing, RDF projection, SHACL validation, pattern materialization, and integration with the graph database and local language-model workflow. Case representations, FCM-FRHP reference structures, pattern templates, detected instances, and rule-application traces were stored and queried in Neo4j 5.26.19 using Cypher. RDF projections and SHACL validation were implemented in Python using rdflib 7.6.0 and pySHACL 0.31.0. Graph visualizations were generated with Cytoscape.js 3.26.0.

Generative AI was used only in the final interpretive stage described above. The model was locally executed through Ollama v0.30.10 (Ollama, 2026) using mistral-small3.2:24b, corresponding to the Mistral Small 3.2 24B instruct model family (Mistral AI, 2025), with fixed inference settings as reported in Section 2.10. All pattern detection, ranking, validation, and materialization steps were rule-based. Generated outputs were treated as structured explanatory summaries requiring expert review.

The complete reproducibility package is publicly archived in Zenodo (Hurtado-Martínez et al., 2026). It includes the full synthetic WimpGrid and derived FCM representation, the FCM-FRHP reference layer and factor-by-pattern affinity settings, pattern templates and role constraints, Cypher queries, RDF/SHACL shapes, prompt and post-processing specifications, notebooks, rule-application traces, candidate-flow and parameter-sensitivity audits, and generated outputs. These materials provide the inputs, configuration, executable procedures, and intermediate evidence required to reproduce the reported rule-based analyses.

## 3. Results

Because the study is methodological in scope, the results are presented in two complementary parts. Section 3.1, Section 3.2, Section 3.3, Section 3.4 and Section 3.5 report the end-to-end application of the pipeline to the illustrative synthetic formulation. This part shows how a WimpGrid-derived PMS-FCM can be represented, structurally qualified, queried, materialized, traced, and interpreted.

Section 3.6 reports the separate controlled synthetic rule-fidelity evaluation. These results compare prespecified and observed outcomes for positive, global-negative, near-miss, and competing-candidate configurations. Neither component constitutes a sample-based inferential evaluation or an estimate of detection performance in clinical case populations.

### 3.1. Illustrative Synthetic Case

To illustrate the proposed workflow, we constructed a synthetic FCM-FRHP case representation based on a fictional idiographic formulation. The case was designed to preserve the structural and semantic properties required for pattern detection while avoiding the use of identifiable or clinically sensitive material. It should therefore be understood as a methodological vignette rather than as a clinical case report. At the same time, the vignette was not intended as an arbitrary artificial example: its construction was informed by recurrent case-formulation issues encountered in research and supervision activities within the Constructivist Research Group at UNED (GICUNED), without being derived from, or corresponding to, any identifiable clinical case.

The formulation was constructed as if the bipolar constructs had been elicited within the FCM-FRHP formulation framework. In this sense, constructs were not treated as generic variables subsequently assigned to external categories. Rather, they were presented as constructs emerging within the functional organization of the FRHP framework itself, where each elicited construct is situated in relation to the formation or resolution of the person’s problem. The resulting representation therefore preserves the intended logic of the FCM-FRHP procedure: idiographic constructs are formulated within clinically meaningful functional positions, while still retaining their bipolar and personal meaning structure.

The vignette describes a person experiencing a state of functional blockage, low activation, and difficulty organizing change. The central problem-oriented construct was represented by the bipolar construct *Paralyzed and disconnected—Able to activate myself*. This difficulty was embedded in a broader context involving unstable routines, poor effort regulation, loss of daily organization, recent work overload, overwhelmed time management, and a tendency to procrastinate. These constructs were presented as part of the problem-formation side of the FCM-FRHP formulation, including problem-oriented, predisposing, triggering, and maintenance-related positions.

The reconstructive side of the formulation included constructs related to active commitment to change, strategic clarity, self-confidence, motivation, perceived self-efficacy, goal clarification, and the ability to manage obstacles. However, the formulation also encoded tensions between these reconstructive directions and several limiting conditions. For example, strategic change was represented as desirable, but potentially constrained by low self-efficacy, confused goals, and obstacles experienced as blocking. The synthetic case was therefore designed to contain both problem-maintaining and reconstruction-oriented relations, allowing the pipeline to detect selected systemic archetype-inspired configurations within a single idiographic graph.

### 3.2. WimpGrid Encoding and FCM-FRHP Representation

The synthetic case was encoded as a Weighted Implication Grid (WimpGrid). In this representation, each bipolar construct was oriented so that the right pole corresponded to the pole identified as desired by the participant through the Ideal-Self position. This convention allowed signed weighted implications to be interpreted as possible movements toward or away from the idiographically desired pole of each construct, rather than as generic increases or decreases in abstract variables.

The WimpGrid was transformed into a weighted directed FCM, where constructs were represented as nodes and weighted implications as directed edges. Each construct was then linked to its corresponding FCM-FRHP functional factor. Table 2 summarizes the construct set used in the synthetic case.

For C11, undefined-self status indicated a central Self-Now position ($s_{11}=0$), meaning that the current self was not clearly identified with either pole. Its non-central Ideal-Self score ($d_{11}=0.70$), oriented toward *Self-confidence*, determined the canonical pole orientation in the same way as for any construct with a uniquely defined Ideal-Self position.

For readability, the complete WimpGrid matrix is not displayed in the main text. Instead, Table 3 reports the WimpGrid-derived weighted implications that directly support the detected pattern instances. The full synthetic WimpGrid matrix and the corresponding computational materials are publicly available in the Zenodo reproducibility repository (Hurtado-Martínez et al., 2026).

**Figure 4:**
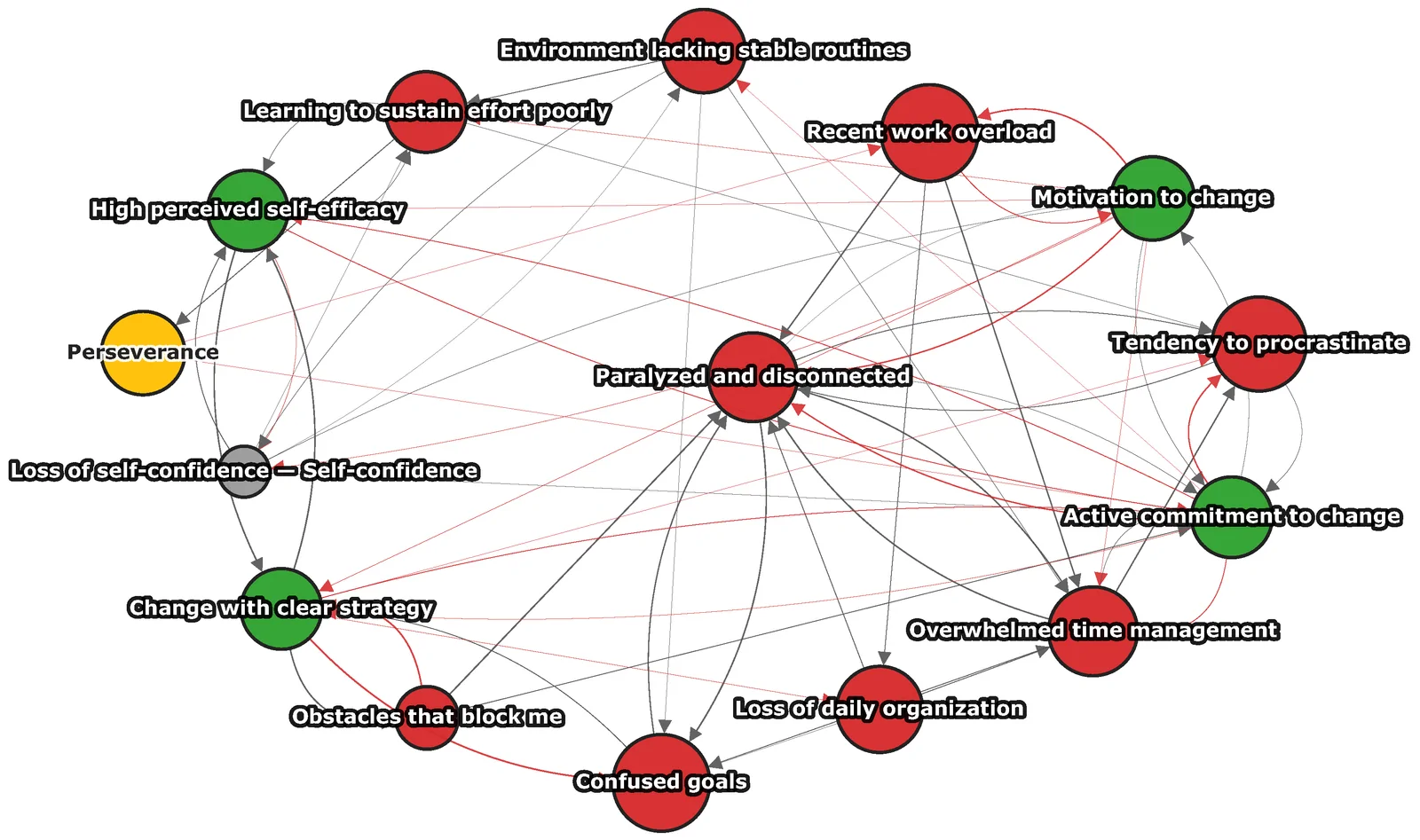
Weighted directed FCM derived from the synthetic WimpGrid. Nodes represent bipolar constructs and directed edges represent signed weighted implications. Black and red edges indicate positive and negative weights, respectively. Node colors denote construct status: red = discrepant, green = congruent, yellow = dilemmatic, and gray = undefined self. Node size represents the absolute self–ideal discrepancy. This graph constitutes the idiographic substrate for PB-based qualification and archetype-inspired pattern detection.

The resulting graph is shown in Figure 4. The figure represents the complete FCM substrate from which the pattern candidates were retrieved.

### 3.3. PB-Based Structural Qualification

The constructs of the synthetic FCM were projected into the Presence–Implication Balance (PB) space as a structural qualification step (Sanfeliciano et al., 2025b). Presence *P* summarizes the overall level of implication of each construct within the graph, whereas Implication Balance *B* represents the asymmetry between outgoing and incoming implication.

In the present results, PB information was used as a limited structural qualifier: it identified comparatively low-Presence constructs for eligibility filtering when required by a pattern template, but did not independently define clinical meaning, assign FCM-FRHP roles, or determine pattern membership.

The shape of the within-map Presence distribution was explored descriptively using the Shapiro–Wilk test. For the 15 constructs in the synthetic PMS-FCM, the test did not indicate a statistically significant departure from normality (W=0.965, p=0.775). Given the small number of constructs, this result was interpreted only as limited descriptive evidence that the observed values were compatible with a normal-shaped distribution.

Independently of this descriptive assessment, the nominal idiographic cutoff was calculated as one sample standard deviation below the within-map mean:

(4) $$P_{\mathrm{cut}}=\bar{P}-s_{P}.$$

With $\bar{P}=0.072$ and $s_{P}=0.040$, this yielded $P_{\mathrm{cut}}=0.032$. The cutoff was treated as a configurable structural-salience criterion rather than as an empirically calibrated clinical or psychometric threshold.

Two constructs were marked as comparatively low in Presence: *Environment lacking stable routines—Environment with stable routines* and *Low tolerance for effort—Perseverance*. These constructs, summarized in Table 4, remained part of the case representation, but they were not prioritized for pattern-role assignment when the corresponding template required low-*P* filtering.

**Figure 5:**
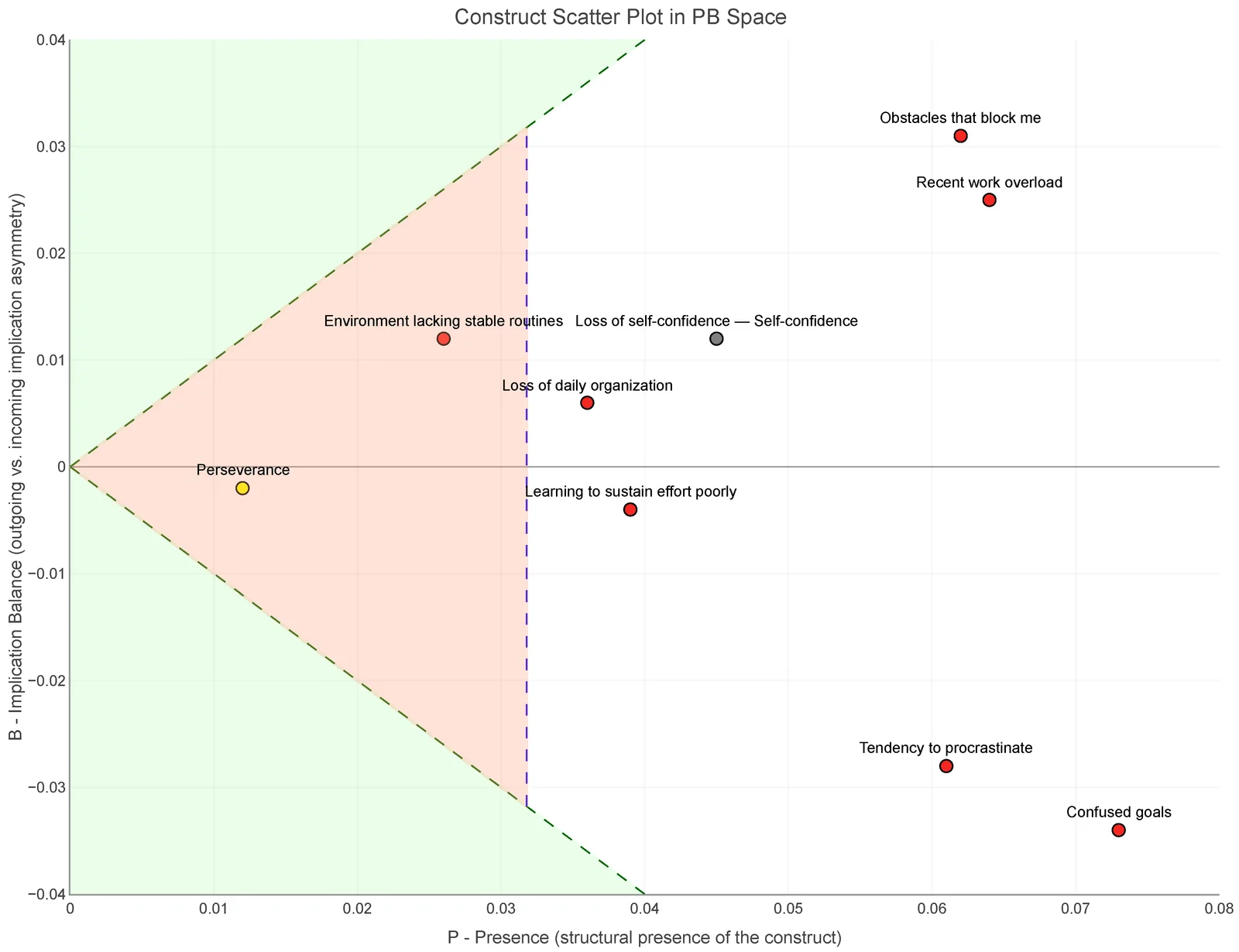
Zoomed Presence–Implication Balance (PB) projection of the synthetic PMS-FCM case around the origin of the PB space. Each point represents a bipolar construct and is labeled by its self pole, except in undefined-self constructs, for which both poles are displayed. The salmon-shaded area indicates the low-presence zone used for structural filtering in the present example. Pale-green shading denotes the non-viable region outside the PB space. Point colors denote construct status: red = discrepant, green = congruent, yellow = dilemmatic, and gray = undefined self.

Figure 5 shows the PB projection of the synthetic case. The plot marks the low-presence region used for structural filtering and labels the constructs included in the zoomed PB-space view. PB information did not determine pattern membership by itself; rather, it acted as an additional structural constraint or qualification criterion within the graph-semantic detection process.

### 3.4. Detected Archetype-Inspired Configurations

Consistent with the methodological scope defined in Section 1.7, the instances reported below are static configurations compatible with the encoded templates. They are presented as rule-conformant outputs of the pipeline, rather than as evidence that the corresponding systemic processes unfolded over time.

The illustrative analysis focused on two pattern templates: Escalation and Limits to Growth. These templates were selected because they were clearly represented in the synthetic FCM-FRHP formulation and because they allowed the pipeline to be illustrated without overloading the example with all available pattern families. The purpose of this section is therefore not to exhaust all possible archetype-inspired configurations, but to show how selected templates can be characterized, detected, materialized, traced, and interpreted within an individual FCM.

Application of the detection pipeline yielded two structurally compatible archetype-inspired configurations: one Escalation instance and one Limits to Growth instance. Both instances were first retrieved through graph queries, ranked according to template-specific criteria, projected into RDF, shown to conform to the encoded SHACL requirements, and finally materialized as explicit pattern nodes in the graph. The two detected pattern instances and their main selection, conformance, and ranking information are summarized in Table 5.

#### 3.4.1. Escalation

The first detected configuration was compatible with the Escalation template. It involved a positive reciprocal coupling between C1, *Paralyzed and disconnected—Able to activate myself*, and C6, *Overwhelmed time management—Sustainable time management*. The former acted as the problem-oriented anchor of the configuration, corresponding to the central distress-related focus of the synthetic formulation, whereas the latter acted as the escalation counterpart.

The semantic-affinity constraint was satisfied because C6 instantiated Maintenance factors, a non-anchor FCM-FRHP factor with sufficient affinity with the Escalation pattern family under the configured reference graph parameters. The counterpart also passed the low-*P* exclusion criterion.

The two required directed relations were both positive and exceeded the minimum edge-weight threshold used by the template. The relation from C1 to C6 had a weight of 0.306, and the reciprocal relation from C6 to C1 had a weight of 0.331. The resulting structural strength was 0.318 and the reciprocity balance was 0.961, producing a ranking score of 0.306. The candidate passed the RDF/SHACL conformance check, and one Escalation instance was materialized.

**Figure 6:**
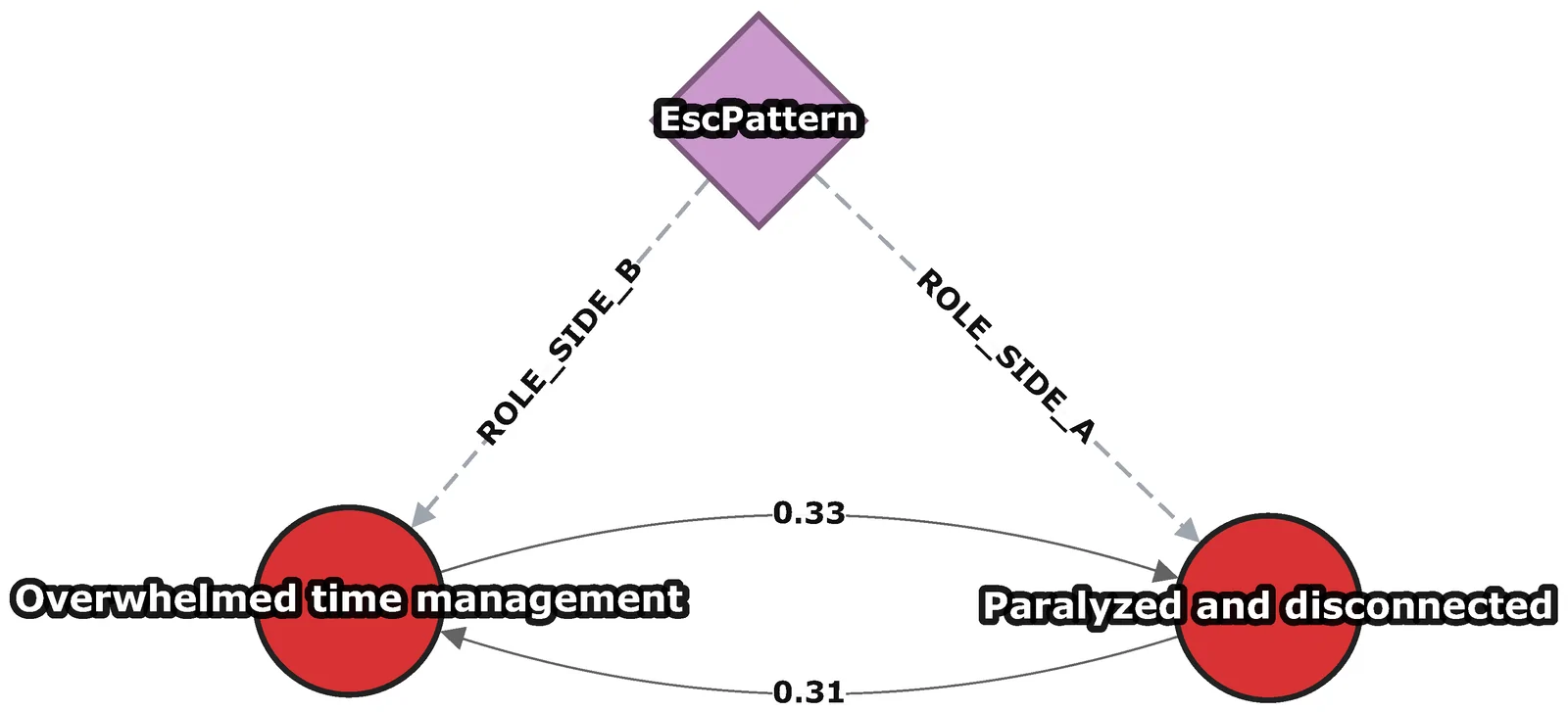
Detected Escalation instance in the synthetic FCM-FRHP case. The materialized pattern node is linked to the constructs participating as SIDE_A and SIDE_B through role-specific relations.

Substantively, this configuration suggests a possible mutually reinforcing relation between functional blockage and overwhelmed time management. If C1 shifts toward its undesirable pole, *paralyzed and disconnected*, it may contribute to C6 also shifting toward its undesirable pole, *overwhelmed time management*. Conversely, if C6 shifts toward overwhelmed time management, it may reinforce C1 moving toward paralysis and disconnection. The materialized instance is shown in Figure 6.

#### 3.4.2. Limits to Growth

The second detected configuration was compatible with the Limits to Growth template. It involved C9, *Change without clear strategy—Change with clear strategy*, as GROWTH; C13, *Low perceived self-efficacy—High perceived self-efficacy*, as ENABLER; and C15, *Obstacles that block me—Obstacles I can manage*, as LIMIT. This configuration combined a positive reinforcing engine between GROWTH and ENABLER with a limiting feedback mechanism through LIMIT.

The semantic-affinity constraints were satisfied because C9 instantiated Reconstruction process, the configured reconstruction-oriented anchor for GROWTH; C13 instantiated Motivation, a factor compatible with enabling reconstructive movement; and C15 instantiated Difficulties, a factor compatible with limiting or blocking reconstructive movement. The GROWTH anchor also passed the low-*P* exclusion criterion.

The positive reciprocal relation between GROWTH and ENABLER was represented by C9 → C13 and C13 → C9. The limiting mechanism was represented by C9 → C15 and C15 → C9, where the feedback from C15 to C9 was negative. In the synthetic case, the enabler strength was 0.362, the limit strength was 0.348, and the final Limits to Growth score was 0.106. The candidate passed the RDF/SHACL conformance check, and one Limits to Growth instance was materialized.

**Figure 7:**
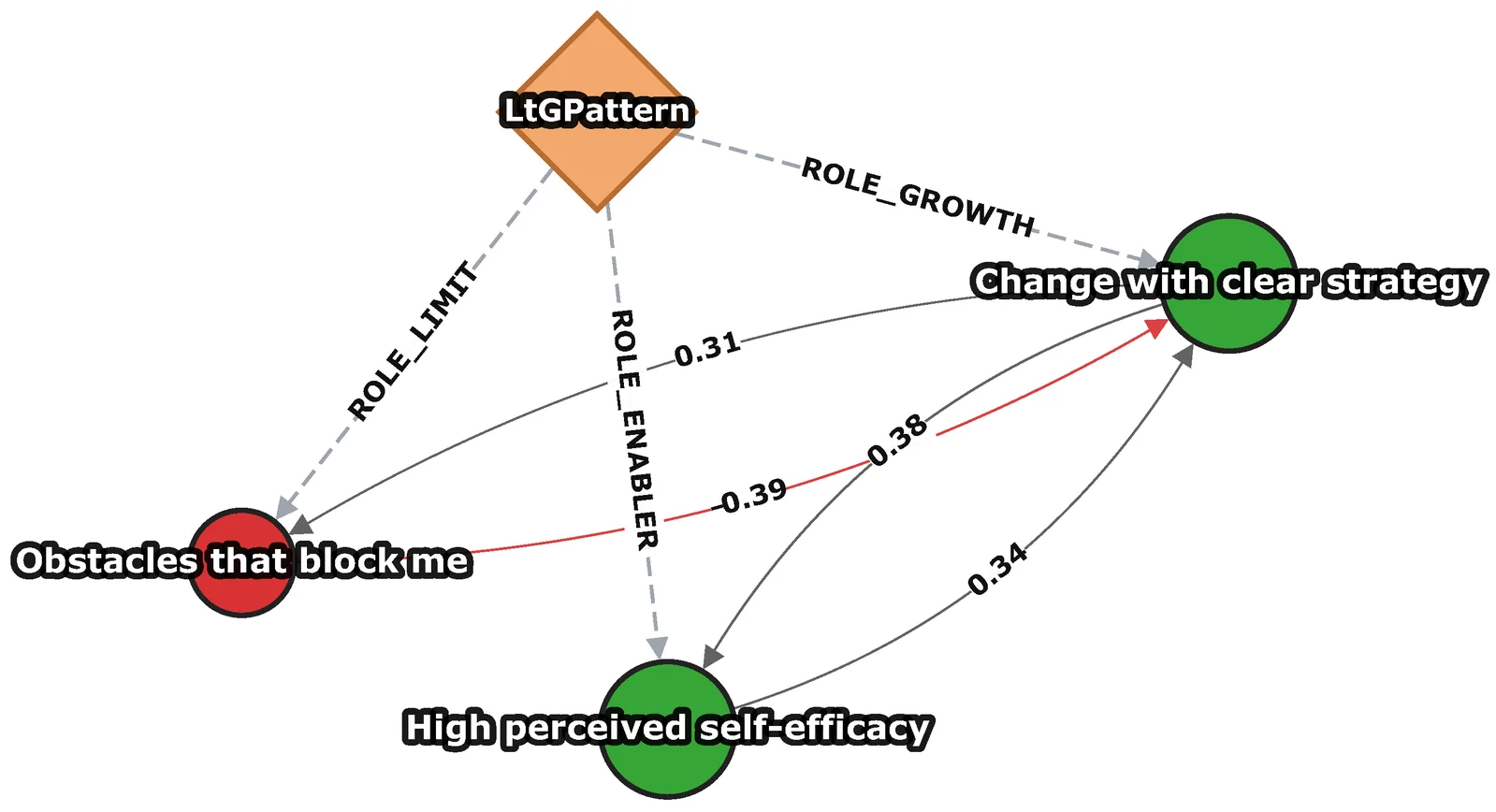
Detected Limits to Growth instance in the synthetic FCM-FRHP case. The materialized pattern node is linked to the constructs instantiating the GROWTH, ENABLER, and LIMIT roles.

Substantively, this configuration illustrates how movement toward clearer and more strategic change may be supported by higher perceived self-efficacy. At the same time, the change process is structurally linked to obstacles, and those obstacles negatively feed back into strategic change. If this structure were maintained or intensified, reconstructive movement could become constrained by the obstacles activated around change. The materialized instance is shown in Figure 7.

#### 3.4.3. Parameter Sensitivity of the Illustrative Detections

The local one-factor-at-a-time audit comprised 93 scenarios across the four pattern templates. The interpretation reported here focuses on Escalation and Limits to Growth because these were the two templates with compatible reference candidates in the illustrative PMS-FCM. The complete scenario-level and candidate-level results are available in the reproducibility package (Hurtado-Martínez et al., 2026) and are summarized in Appendix C, Table A3.

The Escalation reference instance was preserved at minimum edge-weight and structural-strength thresholds of 0.25 and 0.30, but no compatible instance remained when either threshold was increased to 0.35. This boundary effect is consistent with its minimum supporting edge weight of 0.3062 and structural strength of 0.318. The instance was preserved across reciprocity-balance thresholds from 0.60 to 0.90 and counterpart-affinity thresholds from 0.40 to 0.60.

The Limits to Growth reference instance was likewise preserved at minimum edge-weight and component-strength thresholds of 0.25 and 0.30, but not at 0.35. This result is consistent with its minimum supporting edge weight of 0.3103 and limiting-component strength of 0.3485. The instance was preserved at component-balance thresholds of 0.60, 0.70, and 0.80, but not at 0.90, which exceeded its limiting-component balance of 0.8845. Selection remained unchanged across role-affinity thresholds from 0.40 to 0.60.

Across the examined low-P settings (κ=0.5, 1.0, and 1.5) and when the PB filter was disabled, the selected Escalation and Limits to Growth instances remained unchanged. Thus, the two illustrative detections did not depend on the particular low-P setting within the examined range.

Retaining all compatible candidates also produced the same selected set because only one compatible candidate remained for each template; the reference top-*k* settings were therefore non-binding. Alternative multiplicative, arithmetic-mean, and minimum-component score aggregations selected the same candidates, but these comparisons were not informative for ranking sensitivity because only one compatible candidate reached the ranking stage in each template. Overall, the two illustrative detections were stable across the examined affinity and PB settings and across moderate balance variations, but were boundary-sensitive to stricter edge-weight and strength thresholds.

### 3.5. Traceable Pattern Materialization

Both detected instances were materialized as explicit graph nodes and linked to their corresponding pattern templates through INSTANCE_OF_PATTERN_TEMPLATE relationships. Participating constructs were linked to the materialized pattern nodes through role-specific relationships, including ROLE_SIDE_A, ROLE_SIDE_B, ROLE_GROWTH, ROLE_ENABLER, and ROLE_LIMIT. This design made the detected configurations inspectable as part of the graph itself, rather than as external annotations.

Each materialized pattern retained explicit traces of the rule-application process. These traces documented the structural basis of detection, the FCM-FRHP role constraints, the pattern-affinity criterion, the PB-based qualification or filtering when applicable, the ranking criterion, and the role-specific reasons for assigning each construct to its corresponding pattern role. Thus, the output of the pipeline was not simply a pattern label, but a graph-based and auditable representation of why the candidate was retrieved, considered semantically compatible, ranked, shown to conform, and materialized.

The rule-application trace components shown in Table 6 provide selected examples of the trace information generated for each detected pattern instance. These examples should be read in relation to the operational parameters summarized in Table 1. The complete graph-level trace includes additional metadata, role assignments, PB descriptors, structural metrics, template identifiers, validation information, and prompt-related fields.

### 3.6. Controlled Synthetic Rule-Fidelity Results

The controlled suite comprised ten prespecified test specifications distributed across nine synthetic PMS-FCMs and yielded 13 template-level outcomes. All 13 observed outcomes agreed with their prespecified expectations.

The four positive configurations were recovered and materialized as specified, providing one positive instance for each of the Escalation, Limits to Growth, Eroding Goals, and Reinforcing Loops in Competition templates. The global-negative PMS-FCM produced no admissible candidate, selected candidate, SHACL-conformant instance, or materialized pattern under any of the four templates.

The four near-miss configurations were rejected at the intended rule stage. The wrong-sign dyad was rejected by the signed-edge condition. The insufficient-reciprocity configuration retained a reciprocal structural strength of 0.632, but its reciprocity balance of 0.571 fell below the configured threshold of 0.70. The functional-role near miss was rejected because the proposed SIDE_A construct instantiated Predisposing factors rather than the required Problem factor. The low-P near miss was excluded because its SIDE_B construct occupied a role subject to the configured low-P eligibility restriction.

In the competing-candidate case, all five prespecified Escalation candidates satisfied the eligibility conditions. Their ranking scores were 0.7820, 0.6484, 0.5530, 0.4571, and 0.4190. Under the configured top_k_per_problem = 1 rule, the highest-ranked candidate was selected, shown to conform to the encoded SHACL conditions, and materialized. The remaining four admissible candidates were preserved in the operational traces with the terminal reason NOT_SELECTED_BY_RANKING.

Across the active operational runs of the four template pipelines, nine admissible candidates entered the operational ranking stage. Five were selected, all five conformed to the encoded SHACL requirements, and all five were materialized. The review comprised 22 candidate traces, 13 template-level test-case outcomes, and five materialized pattern instances. All aggregate, candidate-level, outcome-level, and trace-count expectations matched, with no execution errors or warnings.

Table 7 summarizes the prespecified expectations and corresponding observed outcomes for the controlled synthetic rule-fidelity evaluation.

### 3.7. Structured Interpretation of the Detected Patterns

After graph-based retrieval, ranking, RDF/SHACL conformance checking, and materialization, the detected pattern instances were submitted to the local language-model workflow described in Section 2.10. At this stage, the model was used only as a constrained graph-to-text layer for producing concise descriptions grounded in the materialized pattern, the participating constructs, their FCM-FRHP roles, and the signed weighted relations.

Because the language-model output was downstream from the rule-based pipeline, the primary result remained the materialized and traceable graph instance. The generated summaries were therefore used only as structured interpretive descriptions of already materialized, SHACL-conformant configurations. Table 8 reports these graph-grounded interpretations in a concise, publication-oriented form.

## 4. Discussion

The illustrative case shows how systemic archetype-inspired configurations can be operationalized as semantically constrained graph structures within FCM-FRHP-enriched idiographic formulations. The pipeline combined FCM-FRHP role semantics, PB-based structural qualification, template-specific ranking, RDF/SHACL conformance checking, and graph materialization. It produced explicit and traceable instances of two pattern-compatible configurations: Escalation and Limits to Growth.

Accordingly, the present study provides a controlled methodological demonstration of technical feasibility and rule fidelity: it shows that predefined graph configurations can be represented, retrieved, ranked, checked for rule conformance, materialized, traced, and rendered as constrained summaries under synthetic conditions. The templates and interpretive constraints were also informed by the authors’ clinical and theoretical experience in constructivist psychotherapy, personal meaning systems, and case formulation, providing relevant domain grounding for their initial operationalization. This expert-informed foundation should nevertheless be distinguished from independent empirical evidence. The study does not establish the construct validity of the archetype-inspired templates, the semantic validity of the functional-role and affinity assignments, their criterion correspondence with independent clinical judgments, inter-rater or test–retest reliability, predictive validity for therapeutic process or outcome, or clinical utility in improving formulation or intervention planning. These levels of evidence require separate empirical evaluation.

### 4.1. From FCM-FRHP Representation to Pattern-Level Analysis

A first contribution of the proposed approach is that it extends the analytical use of FCM-FRHP beyond the representation of individual constructs and pairwise implications. The FCM-FRHP framework already provides a structured way to represent idiographic case formulations through fuzzy cognitive maps, preserving both the individualized character of clinical formulation and the possibility of analyzing weighted directed relations between constructs (Botella, 2024; Botella et al., 2022; Saúl et al., 2023). The present work adds a further layer: the detection of higher-order configurations defined as semantically constrained subgraphs.

This extension is coherent with the logic of case formulation as more than a list of relevant variables. In clinical practice, formulation requires the identification of organized patterns of interaction among problems, maintaining factors, reconstructive processes, resources, motivations, and obstacles (Eells, 2015; Kuyken et al., 2009; Persons, 2008). By formalizing such configurations as graph templates, the proposed pipeline makes it possible to inspect whether a given idiographic FCM contains structures compatible with systemic organization, while preserving the construct-level specificity of the case.

Crucially, the framework does not assign archetype labels to an FCM on the basis of structural resemblance alone. Candidate subgraphs must satisfy the template’s configured functional-role, semantic-affinity, polarity, signed-edge, structural, and, where applicable, PB-based eligibility conditions. Only candidates retained after template-specific ranking and shown to conform to the encoded SHACL rules are materialized as template-compatible instances. This makes the attribution explicit and auditable, without treating rule conformance as independent evidence of the construct or clinical validity of the template.

The detected patterns should not be understood as universal clinical categories. Rather, they are template-compatible graph configurations whose meaning depends on the concrete bipolar constructs involved, their self–ideal orientation, their FCM-FRHP functional roles, their semantic affinity with the pattern family, and their position within the individual network. This is particularly important in the context of personal construct approaches, where constructs are not generic variables but bipolar dimensions embedded in an idiographic meaning system (Botella & Feixas, 1998/2008; Feixas & Villegas, 1990; Kelly, 1955).

### 4.2. Reinterpreting Systemic Archetypes in Intrapersonal Cognitive Systems

The second contribution concerns the reinterpretation of systemic archetypes for intrapersonal cognitive systems. Classical systemic archetypes describe recurrent structural logics associated with behavior over time in complex systems (Meadows, 2008; Senge, 2006; Sterman, 2000). However, they were not originally formulated for idiographic psychological case formulations represented as bipolar construct systems. Therefore, their direct transfer to FCM-FRHP would be conceptually problematic.

The present approach addresses this issue by treating archetype-inspired patterns as constrained graph templates rather than as literal imports of classical archetypes. For example, an Escalation instance is not interpreted as proof that a person is undergoing an observed escalation process over time. It is interpreted as a static structure compatible with reciprocal reinforcement between a problem-oriented construct and an eligible counterpart. Similarly, a Limits to Growth instance is not treated as evidence of a temporal trajectory of growth followed by plateau or decline. It is interpreted as a structure in which a reconstruction-oriented process is supported by an enabler while also being constrained by a limiting feedback relation.

Consistent with the methodological scope defined in Section 1.7, the detected patterns are interpreted as template-compatible static structures. Examining their correspondence with psychological processes unfolding over time requires longitudinal or repeated FCM-FRHP assessments.

### 4.3. Traceability, Rule Application, and Methodological Control

A third contribution is the emphasis on traceable materialization. The pipeline does not merely assign a label indicating that a pattern has been detected. Instead, each detected instance is materialized as a graph node linked to its template, its role definitions, and its participating constructs. The detection process also preserves traces documenting the structural conditions, FCM-FRHP role constraints, semantic-affinity criterion, PB-based qualification, ranking criteria, and validation outcome.

This design supports methodological transparency in several ways. First, it makes explicit why a candidate was retrieved and retained. Second, it separates the different sources of admissibility: semantic compatibility, structural topology, edge sign and magnitude, FCM-FRHP role compatibility, PB-based qualification, and ranking. Third, it allows the detected pattern to be inspected as part of the same graph environment as the original FCM-FRHP case representation. In this sense, the result is not an external annotation imposed after the fact, but an auditable graph object generated through explicit rules.

This point also clarifies the role of generative AI in the workflow. The local language model was not used for pattern discovery, role assignment, ranking, or validation. These operations were handled by graph querying, semantic-affinity constraints, structural constraints, and RDF/SHACL conformance checking. The language model was used only downstream, as a constrained graph-to-text layer for producing structured interpretations of already materialized pattern instances. This separation reduces the risk of interpretive drift and preserves the primacy of rule-based detection.

### 4.4. Role of PB-Based Structural Qualification

PB-space information provided an additional structural qualification layer within the pipeline. Presence (P) and Implication Balance (B) allowed constructs to be positioned according to their implication profile within the FCM (Sanfeliciano et al., 2025b). In the illustrative case, the PB projection was used to identify comparatively low-presence constructs for structural filtering purposes where configured, and to support the prioritization of structurally salient candidates.

This use of PB information is intentionally limited. PB-based qualification does not determine clinical meaning by itself, nor does it assign FCM-FRHP roles or define pattern membership independently. Instead, it functions as one component of a broader graph-semantic detection process. Depending on the template, PB information may operate either as an ex ante eligibility filter or as a posterior structural qualifier, since low-presence constructs may still be clinically informative in some pattern configurations.

The operational low-*P* cutoff used in the synthetic example should be understood as a design-based structural-salience criterion rather than as a clinical or psychometric threshold. The sensitivity analysis examined how candidate eligibility, ranking, and pattern materialization responded to plausible variations in κ and to disabling the filter. These comparisons addressed computational robustness to the selected design parameter; they did not empirically calibrate the cutoff or establish its clinical validity. Further empirical work should determine whether alternative PB-based thresholds or case-specific calibration procedures produce stable and clinically useful pattern detection.

### 4.5. Interpretation of the Illustrative Case

The synthetic case was designed to contain two clearly interpretable configurations. The Escalation instance linked the problem-oriented construct *Paralyzed and disconnected—Able to activate myself* with *Overwhelmed time management—Sustainable time management*. This reciprocal coupling suggests a possible structure in which functional blockage and overwhelmed time management could reinforce movement toward their undesirable poles. The Limits to Growth instance linked *Change without clear strategy—Change with clear strategy*, *Low perceived self-efficacy—High perceived self-efficacy*, and *Obstacles that block me—Obstacles I can manage*. This configuration suggests a reconstruction-oriented pathway in which strategic change may be supported by perceived self-efficacy while also being constrained by obstacles that negatively feed back into the change process.

These graph-grounded interpretations illustrate how the pipeline can represent, detect, trace, and support a structured reading of selected systemic configurations in the synthetic formulation. Their correspondence with psychological processes unfolding over time remains an empirical question to be examined through longitudinal or repeated FCM-FRHP assessments.

In applied case-formulation work, such a Limits-to-Growth-compatible configuration could be used as a prompt for collaborative inquiry rather than as a clinical conclusion. In the present example, the clinician might explore with the client how clearer strategic change and perceived self-efficacy support one another, which obstacles become activated when change is attempted, and how those obstacles may constrain the reconstructive process. The detected pattern would therefore function as a structured conversation aid for reviewing and refining the formulation, rather than as an automated diagnosis or treatment recommendation.

### 4.6. Limitations

Several limitations should be noted. First, the proposed approach was evaluated using a synthetic case rather than real clinical data. This enabled controlled illustration of the full pipeline and avoided disclosure of sensitive material, but restricts conclusions about clinical validity, real-world prevalence, reliability, and practical usefulness. The controlled synthetic suite extends the evaluation beyond the engineered success case by including positive, global-negative, single-condition near-miss, and competing-candidate fixtures. Nevertheless, the suite was designed to examine rule fidelity rather than to estimate detection performance. The cases were prespecified and structurally controlled, and the near-miss perturbations were concentrated on the Escalation template because its compact reciprocal structure facilitated isolation of individual rules. Larger and more heterogeneous synthetic and empirical case sets will be required to estimate false-positive and false-negative rates and to evaluate generalization beyond the encoded fixtures. Second, the detected patterns were examined in a static FCM representation; longitudinal or repeated FCM-FRHP assessments will be needed to test whether these configurations correspond to actual trajectories of problem maintenance or therapeutic change. Third, although the reproducibility package includes notebooks for detecting all four pattern templates, only one synthetic case and two detected templates, Escalation and Limits to Growth, were presented in detail in the article. This preserved clarity, but future work should evaluate Eroding Goals and Reinforcing Loops in Competition across broader synthetic and empirical datasets. Fourth, the pipeline depends on explicit design choices, including edge-weight thresholds, pattern-affinity parameters, PB-based filters, and ranking formulas. The local OFAT analysis showed that the two illustrative detections were preserved across the examined affinity and PB settings and across moderate balance variations, but were removed by stricter edge-weight or strength thresholds. These results identify boundary sensitivity in the illustrative configurations rather than establishing general parameter robustness. Because the analysis varied one parameter at a time within one synthetic PMS-FCM, broader multivariable sensitivity analyses, empirical calibration, and external evaluation remain required. Fifth, the implementation combines property-graph queries for candidate retrieval with RDF/SHACL conformance checking. This supports explicitness, auditability, and separation of concerns, but should be compared with simpler property-graph-only implementations. Finally, the language-model interpretation layer was used only as a controlled narrative rendering layer over already rule-conformant graph instances. Although prompts were grounded in graph evidence and outputs were stored with model and prompt-trace metadata, the resulting interpretations should be treated as graph-grounded summaries requiring expert review, not as autonomous clinical conclusions.

### 4.7. Future Directions

Subsequent studies should move from methodological illustration toward systematic validation. Priority directions include evaluating the reliability of pattern detection across multiple synthetic and empirical FCM-FRHP cases, testing whether static pattern-compatible structures correspond to longitudinal changes in repeated formulations, and analyzing the robustness of detection under alternative edge-weight thresholds, pattern-affinity parameters, PB-based filters, and ranking formulas. The factor-by-pattern affinity configuration should also be subjected to structured external review by independent clinicians and methodologists with complementary expertise in constructivist case formulation, FCM-based modeling, and systemic archetypes, using standardized rating criteria, inter-rater agreement analysis, and consensus-based revision. Another relevant extension would be to examine whether semantic-embedding representations of idiographic constructs can complement the current factor-level affinity parameters by estimating the semantic proximity between construct meanings and pattern-role semantics. These analyses would help determine which parameters can remain configurable and which may require empirical calibration before broader clinical or research use.

Further work should also examine clinical interpretability and practical usefulness. Independent clinicians with relevant expertise could assess whether detected configurations provide meaningful reformulations of case structure, whether the traces are understandable, and whether graph-grounded summaries support rather than distort clinical reasoning. The graph schema may also be extended to represent multiple time points, alternative formulations, therapist–patient revisions of the same case, or embedding-based semantic qualifications of construct roles, allowing pattern detection to be studied as part of an evolving formulation process rather than as a single static analytic output.

## 5. Conclusions

This article presented a semantically constrained, graph-based methodology for detecting systemic archetype-inspired configurations in idiographic FCM-FRHP case representations. The approach reformulates selected systemic archetypes as configurable graph templates adapted to intrapersonal bipolar construct systems, combining FCM-FRHP semantics, signed weighted relations, PB-based structural qualification, template-specific ranking, RDF/SHACL conformance checking, and traceable graph materialization.

The synthetic case illustrated how two pattern-compatible configurations, Escalation and Limits to Growth, can be detected and interpreted as static graph structures rather than as clinical facts or observed temporal processes. A central contribution of the framework is its traceability: detected patterns remain linked to their templates, roles, participating constructs, semantic criteria, structural evidence, and rule-application traces.

Overall, this study provides a controlled and reproducible methodological implementation connecting constructivist case formulation, graph-based modeling, and systemic theory under synthetic conditions. Its clinically and theoretically informed design provides a basis for subsequent empirical evaluation of construct and semantic validity, reliability, correspondence with independent clinical judgments, predictive validity, clinical interpretability, longitudinal relevance, and potential utility for psychological case formulation and intervention planning.

## Figures and Tables

**Table 1:** Operational parameters retrieved from the pattern-template nodes and used in the illustrative synthetic case analysis.

Template	Parameter Group	Values	Role in the Pipeline
Escalation	Edge-weight threshold	min_edge_weight = 0.30	Minimum positive weight required for each directed relation in the reciprocal dyad.
Escalation	Structural thresholds	min_structural_strength = 0.30; min_reciprocity_balance = 0.70	Minimum reciprocal strength and balance required for an Escalation-compatible dyad.
Escalation	Semantic-affinity threshold	min_counterpart_affinity = 0.50	Minimum Escalation affinity required for the affinity-filtered SIDE_B role, in addition to exclusion of the Problem anchor factor.
Escalation	Candidate retention	top_k_per_problem = 1; top_k_per_fcm = 5	Number of top-ranked candidates retained per Problem anchor and per PMS-FCM.
Escalation	PB-based filtering	Low-*P* exclusion: $P_{i}<P_{\mathrm{cut}}$	Low-Presence SIDE_B candidates were excluded before ranking.
Limits to Growth	Edge-weight threshold	min_edge_weight = 0.30	Minimum absolute signed edge weight required for the positive growth engine and the limiting feedback mechanism.
Limits to Growth	Enabler thresholds	min_enabler_strength = 0.30; min_engine_balance = 0.70	Minimum strength and balance required for the positive reciprocal growth engine.
Limits to Growth	Limit thresholds	min_limit_strength = 0.30; min_limit_balance = 0.70	Minimum strength and balance required for the limiting feedback mechanism.
Limits to Growth	Semantic-affinity thresholds	min_enabler_affinity = 0.50; min_limit_affinity = 0.50	Minimum Limits to Growth affinity required for the ENABLER and LIMIT roles, jointly with their role-specific functional-factor constraints.
Limits to Growth	Candidate retention	top_k_per_growth = 1; top_k_per_fcm = 5	Number of top-ranked triads retained per Reconstruction process anchor and per PMS-FCM.
Limits to Growth	PB-based filtering	Low-*P* exclusion: $P_{i}<P_{\mathrm{cut}}$	Low-Presence GROWTH candidates were excluded before ranking.
Both illustrated templates	Rule-conformance checking	validation_method = RDF_SHACL	RDF/SHACL conformance checking applied after candidate retrieval, eligibility filtering, and ranking.

Note. Parameters were stored as properties of the corresponding PatternTemplate nodes and loaded at runtime. PB-based filtering is reported according to its analytical meaning, namely the exclusion from template-specific roles of constructs below the case-specific Presence cutoff $P_{\mathrm{cut}}$, rather than according to internal implementation labels. Fixed anchor roles were defined through exact FCM-FRHP factor requirements. Affinity-filtered roles had to satisfy both the reported pattern-specific affinity threshold and the corresponding role-specific functional-factor constraints; reaching the affinity threshold was therefore necessary but not sufficient for role eligibility. Top-*k* parameters controlled candidate prioritization and retention rather than template compatibility. All values are operational configuration settings used for reproducibility and traceability in the synthetic case analysis, not empirically calibrated clinical or psychometric thresholds.

**Table 2:** Synthetic WimpGrid constructs used to instantiate the illustrative FCM-FRHP case.

Pos.	Left Pole	Right Pole	Functional Factor	Construct Status
1	Paralyzed and disconnected	Able to activate myself	Problem	Discrepant
2	Environment lacking stable routines	Environment with stable routines	Predisposing factors	Discrepant
3	Learning to sustain effort poorly	Learning to sustain effort well	Predisposing factors	Discrepant
4	Loss of daily organization	Recovery of daily organization	Triggers	Discrepant
5	Recent work overload	Greater work-life balance	Triggers	Discrepant
6	Overwhelmed time management	Sustainable time management	Maintenance factors	Discrepant
7	Tendency to procrastinate	Continuous engagement	Maintenance factors	Discrepant
8	Low commitment to change	Active commitment to change	Reconstruction process	Congruent
9	Change without clear strategy	Change with clear strategy	Reconstruction process	Congruent
10	Low tolerance for effort	Perseverance	Resources and competencies	Dilemmatic
11	Loss of self-confidence	Self-confidence	Resources and competencies	Undefined self
12	Lack of motivation to change	Motivation to change	Motivation	Congruent
13	Low perceived self-efficacy	High perceived self-efficacy	Motivation	Congruent
14	Confused goals	Clear goals	Difficulties	Discrepant
15	Obstacles that block me	Obstacles I can manage	Difficulties	Discrepant

**Table 3:** Selected WimpGrid-derived weighted implications supporting the detected pattern instances. Construct codes refer to Table 2.

Pattern	Source	Weight	Target	Template Role
Escalation	C1	0.3062	C6	SIDE_A → SIDE_B
Escalation	C6	0.3312	C1	SIDE_B → SIDE_A
Limits to Growth	C9	0.3793	C13	GROWTH → ENABLER
Limits to Growth	C13	0.3448	C9	ENABLER → GROWTH
Limits to Growth	C9	0.3103	C15	GROWTH → LIMIT
Limits to Growth	C15	−0.3913	C9	LIMIT → GROWTH

Note. C1 = *Paralyzed and disconnected—Able to activate myself*; C6 = *Overwhelmed time management—Sustainable time management*; C9 = *Change without clear strategy—Change with clear strategy*; C13 = *Low perceived self-efficacy—High perceived self-efficacy*; C15 = *Obstacles that block me—Obstacles I can manage*. Edge weights are reported with four decimal places; edge labels in figures are rounded for readability. The arrow → indicates the direction of the weighted implication from source to target.

**Table 4:** Low-presence constructs identified for structural filtering in the synthetic case.

Construct	*P*	*B*	Structural Qualification
Environment lacking stable routines—Environment with stable routines	0.026	0.012	Retained in the case graph; excluded from roles subject to the low-presence filter.
Low tolerance for effort—Perseverance	0.012	−0.002	Retained in the case graph; excluded from roles subject to the low-presence filter.

Note. The low-presence cutoff was $P_{\mathrm{cut}}=0.032$, defined as $P_{\mathrm{cut}}=\bar{P}-s_{P}$. Constructs with $P<P_{\mathrm{cut}}$ were marked for structural filtering.

**Table 5:** Detected archetype-inspired pattern instances in the synthetic FCM-FRHP case. Construct codes refer to Table 2.

Pattern	Participating Constructs	Selection and Conformance	Score
Escalation	C1 ↔ C6	Cypher retrieval; semantic-affinity and low-*P* eligibility; dyadic ranking; RDF/SHACL conformance checking.	0.306
Limits to Growth	C9 as GROWTH; C13 as ENABLER; C15 as LIMIT	Cypher retrieval; semantic-affinity and low-*P* eligibility; triadic ranking; RDF/SHACL conformance checking.	0.106

Note. C1 = *Paralyzed and disconnected—Able to activate myself*; C6 = *Overwhelmed time management—Sustainable time management*; C9 = *Change without clear strategy—Change with clear strategy*; C13 = *Low perceived self-efficacy—High perceived self-efficacy*; C15 = *Obstacles that block me—Obstacles I can manage*. The bidirectional arrow ↔ denotes reciprocal coupling between the participating constructs.

**Table 6:** Selected rule-application trace components for the detected pattern instances.

Pattern	Trace Component	Trace Information
Escalation	Pattern summary	Static structure compatible with the Escalation archetype-inspired pattern (positive reciprocal dyad), materialized under template ESCALATION_V1.
Escalation	Structural trace	Escalation candidate: *“Paralyzed and disconnected—Able to activate myself”* and *“Overwhelmed time management—Sustainable time management”* form a positive reciprocal dyad with $w_{ab}=0.306$, $w_{ba}=0.331$, structural_strength=0.318, and reciprocity_balance=0.961.
Escalation	Ranking score	0.305964
Limits to Growth	Pattern summary	Static structure compatible with the Limits to Growth archetype-inspired pattern (growth–enabler–limit triad), materialized under template LIMITS_TO_GROWTH_V1.
Limits to Growth	Structural trace	Limits to Growth candidate: GROWTH and ENABLER form a positive reciprocal growth engine with $w_{ge}=0.3793$, $w_{eg}=0.3448$, enabler_strength=0.3616, and enabler_balance=0.9524; GROWTH and LIMIT form a limiting feedback mechanism with $w_{gl}=0.3103$, $w_{lg}=-0.3913$, limit_strength=0.3485, and limit_balance=0.8845.
Limits to Growth	Ranking score	0.106155

**Table 7:** Expected and observed outcomes of the controlled synthetic rule-fidelity evaluation.

Test Category	Prespecified Expectation	Observed Outcome	Match
Positive recovery	One admissible and materialized instance for each of the four templates.	Escalation, Limits to Growth, Eroding Goals, and Reinforcing Loops in Competition each produced one positive materialized instance.	Yes
Global negative	No admissible or materialized instance under any template.	The PMS-FCM was evaluated independently against all four templates. No candidate was selected, shown to conform to the SHACL requirements, or materialized.	Yes
Wrong-sign near miss	Reject the reciprocal dyad because the required edge signs are incompatible.	The candidate was rejected at the signed-edge stage (WRONG_SIGN).	Yes
Insufficient reciprocity	Reject the candidate because reciprocity balance is below 0.70, despite adequate structural strength.	Structural strength was 0.632, whereas reciprocity balance was 0.571; the candidate was rejected at the structural-balance stage (INSUFFICIENT_BALANCE).	Yes
Ineligible functional role	Reject the candidate because SIDE_A does not instantiate the required Problem factor.	SIDE_A instantiated Predisposing factors; the candidate was rejected at the role-eligibility stage (INELIGIBLE_ROLE).	Yes
Low-P exclusion near miss	Reject the candidate because SIDE_B occupies a low-P-sensitive role and is classified as low in Presence.	The otherwise structurally admissible candidate was rejected at the PB-eligibility stage (LOW_P).	Yes
Competing candidates	Retain and rank all five admissible candidates; select only the highest-ranked candidate under the configured top-*k* rule.	The scores were 0.7820, 0.6484, 0.5530, 0.4571, and 0.4190. The first candidate was materialized; the remaining four were retained in the traces as NOT_SELECTED_BY_RANKING.	Yes

Note. The ten prespecified test specifications yielded 13 template-level outcomes because the global-negative PMS-FCM was evaluated independently against all four templates. Across the active operational runs, nine admissible candidates yielded five selected, SHACL-conformant, and materialized pattern instances. SHACL conformance denotes compliance with the encoded template and data-shape requirements, not independent psychological or clinical validation.

**Table 8:** Graph-grounded structured interpretations of the detected pattern instances.

Pattern	Structured Interpretation
Escalation	This Escalation-compatible pattern is centered on a reciprocal influence between the constructs *“Paralyzed and disconnected—Able to activate myself”* (Problem) and *“Overwhelmed time management—Sustainable time management”* (Maintenance factors). The construct *“Paralyzed and disconnected—Able to activate myself”* may shift toward its undesirable pole of *“Paralyzed and disconnected”*, which could foster a similar shift in *“Overwhelmed time management—Sustainable time management”* toward its undesirable pole of *“Overwhelmed time management”*. If this influence is reciprocal, both shifts may mutually reinforce each other. […]
Limits to Growth	This Limits-to-Growth-compatible configuration is anchored on *“Change without clear strategy—Change with clear strategy”* as a Reconstruction process construct, which forms a positive reciprocal growth engine with the ENABLER *“Low perceived self-efficacy—High perceived self-efficacy”*. The GROWTH construct *“Change without clear strategy—Change with clear strategy”* may shift toward its desirable pole of *“Change with clear strategy”*, while the ENABLER construct *“Low perceived self-efficacy—High perceived self-efficacy”* could move toward its desirable pole of *“High perceived self-efficacy”*. This positive reinforcing loop might support a reconstruction process. However, the GROWTH construct also activates the LIMIT construct *“Obstacles that block me—Obstacles I can manage”*, which negatively feeds back into it. […]

## Data Availability

The complete reproducibility package is publicly available in Zenodo at Hurtado-Martínez et al. (2026). It includes the complete illustrative and controlled synthetic WimpGrid input files and their derived PMS-FCM representations, the case and test manifests, prespecified expected outcomes, the FCM-FRHP reference and semantic-affinity configuration, pattern templates and role constraints, source code, Neo4j/Cypher queries, RDF/SHACL shapes, candidate-level and aggregate results, rule-application traces, materialized pattern records, prompt and post-processing specifications, generated outputs, and the interactive Cytoscape.js visualization. No empirical clinical datasets were used.

## Appendix A. Guiding Questions for the Functional Organization of Construct Positions

The synthetic representations followed a prospectively specified 15-position organization in which construct positions were associated with the eight functional factors of the FCM-FRHP framework. Table A1 presents the guiding questions used to clarify the functional domain represented by each position.

**Table A1:** Author-developed guiding questions and prospective correspondence between construct positions and FCM-FRHP functional factors in the synthetic representations.

Construct Position(s)	FCM-FRHP Functional Factor	Guiding Question
C01	Problem	What is the principal source of psychological dissatisfaction, related to the person’s habits, personality, or values, that they would like and consider possible to change?
C02–C03	Predisposing factors	What factors in the person’s past may have predisposed them to experience this problem or the discrepancy between how they currently are and how they would like to be?
C04–C05	Triggers	What recent factors or events may have activated or precipitated this problem or dissatisfaction?
C06–C07	Maintenance factors	What currently contributes to maintaining the problem or makes it more difficult to resolve?
C08–C09	Reconstruction process	What is the person currently doing to reduce, resolve, or reconstruct the problem situation?
C10–C11	Resources and competencies	What personal resources or competencies support the person’s efforts to reduce or resolve the problem?
C12–C13	Motivation	What motivational processes support the person’s engagement in reducing or resolving the problem?
C14–C15	Difficulties	What conditions, obstacles, or limitations work against the person’s efforts to reduce or resolve the problem?

Note. These guiding questions are included to clarify the intended functional meaning of the FCM-FRHP factors and the prospective correspondence between the 15 construct positions and their functional metadata. They describe the functional organization used to structure the synthetic representations in the present study rather than a separate assessment component evaluated here.

## Appendix B. Pattern-Affinity Configuration

Table A2 reports the complete author-defined factor-by-pattern affinity configuration used in the present implementation. The values encode theory-informed, ordered compatibility judgments used for semantic eligibility and traceability. They should not be interpreted as empirical probabilities, interval-scaled measurements, or independently validated semantic coefficients. The same configuration is available in machine-readable form in the documented implementation archived in Zenodo (Hurtado-Martínez et al., 2026).

**Table A2:** Author-defined factor-by-pattern affinity configuration used in the present implementation.

FCM-FRHP Factor	Esc	LtG	EG	RLC	Compact Functional Rationale
Problem	1.0	0.0	0.9	0.9	Provides the direct problem anchor and represents the central problem-oriented function. It is highly compatible with amplification, erosion, and problem-maintaining loops, but has no operationally permitted role in the current Limits to Growth template.
Predisposing factors	0.4	0.0	0.0	0.0	Represents distal vulnerability or contextual conditions rather than a direct maintaining, reconstructive, limiting, or eroding process. It may have limited indirect compatibility with Escalation, but has no operationally permitted role in the other current templates.
Triggers	0.6	0.0	0.0	0.0	Activates or precipitates the problem and may participate as a problem-related counterpart in amplification. It therefore has moderate compatibility with Escalation, but no operationally permitted role in the other current templates.
Maintenance factors	0.9	0.9	0.9	0.9	Directly sustains the problem and is therefore strongly compatible with amplifying, limiting, eroding, and problem-maintaining loop functions.
Difficulties	0.9	1.0	0.8	0.9	Blocks reconstruction and has direct compatibility with the LIMIT role, as well as strong compatibility with blocking, eroding, and problem-maintaining configurations.
Reconstruction process	0.2	1.0	0.7	1.0	Represents the core reconstructive and growth-oriented function. It is directly compatible with the growth engine and the adaptive loop, may occupy the protective position in Eroding Goals, and has low compatibility with problem-centered Escalation.
Resources and competencies	0.2	0.9	0.7	0.9	Supports reconstruction and protection, making it strongly compatible with growth-supporting and adaptive-loop functions and moderately compatible with the protective component of Eroding Goals.
Motivation	0.2	0.9	0.8	0.9	Supports engagement in reconstruction and movement toward desired positions, with strong compatibility with enabling, protective, and adaptive-loop functions.

Note. Esc = Escalation; LtG = Limits to Growth; EG = Eroding Goals; RLC = Reinforcing Loops in Competition. The numerical values encode an ordered compatibility scheme used in the present implementation: 1.0 denotes core or direct compatibility; 0.8–0.9, strong compatibility; 0.6–0.7, moderate compatibility; 0.5, threshold-level compatibility; 0.4, limited compatibility; 0.2, low compatibility; and 0.0, absence of operational compatibility with any role permitted by the current template. These categories are not assumed to constitute an interval-scaled measure, and differences between adjacent values are not interpreted as equal quantitative units. The affinity matrix encodes factor-to-pattern compatibility, whereas the role-specific allowed-factor constraints encode factor-to-role admissibility. Accordingly, positive affinity with a pattern does not imply eligibility for every role within that pattern. All affinity-filtered roles used a minimum threshold of 0.50, applied jointly with the corresponding role-specific functional-factor constraints. Reaching the numerical threshold was therefore necessary but not sufficient for role eligibility, and lowering the affinity threshold does not override the role-specific constraints. Affinity values were used for semantic eligibility and traceability and were not incorporated into the structural ranking scores. The same values are stored as executable properties of the FCM-FRHP reference nodes in the archived implementation (Hurtado-Martínez et al., 2026).

## Appendix C. Parameter-Sensitivity Summary

Table A3 summarizes the local OFAT results for the two pattern instances materialized in the illustrative PMS-FCM. The complete scenario-level and candidate-level audit is available in the archived implementation (Hurtado-Martínez et al., 2026).

**Table A3:** Local parameter-sensitivity results for the two illustrative pattern instances.

Parameter	Examined Settings	Escalation	Limits to Growth
Minimum edge weight	0.25,0.30,0.35	Reference selection preserved at 0.25 and 0.30; no compatible candidate at 0.35.	Reference selection preserved at 0.25 and 0.30; no compatible candidate at 0.35.
Strength threshold	0.25,0.30,0.35	Reference selection preserved at 0.25 and 0.30; no compatible candidate at 0.35.	Reference selection preserved when both component thresholds were 0.25 or 0.30; no compatible candidate at 0.35.
Balance threshold	0.60,0.70,0.80,0.90	Reference selection preserved at all examined settings.	Reference selection preserved at 0.60, 0.70, and 0.80; no compatible candidate at 0.90.
Affinity threshold	0.40,0.50,0.60	Reference selection preserved at all examined settings.	Reference selection preserved at all examined settings.
PB-based qualification	κ=0.5,1.0,1.5; filter disabled	Reference selection preserved under all four conditions.	Reference selection preserved under all four conditions.
Candidate retention	Reference top-*k*; all compatible	Same selected candidate; top-*k* was non-binding.	Same selected candidate; top-*k* was non-binding.
Score aggregation	Multiplicative; arithmetic mean; minimum component	Not informative for comparative ranking because only one compatible candidate reached this stage.	Not informative for comparative ranking because only one compatible candidate reached this stage.

Note. “Reference selection preserved” indicates that the same candidate selected under the reference configuration remained selected in the varied scenario. The analysis varied one parameter at a time and does not assess interactions among parameters or provide empirical calibration. Top-*k* controls prioritization and materialization, not template compatibility.
